# Plant-Derived Natural Compounds and Nrf2-Centered Redox Signaling in Intracerebral Hemorrhage: Evidence Grading, Mechanistic Boundaries, and Translational Challenges

**DOI:** 10.3390/antiox15070878

**Published:** 2026-07-15

**Authors:** Xu Gao, Yuhao Chang, Yaxin Liu, Ping Tang, Tong Chen, Yue Yuan, Ping Li

**Affiliations:** 1 Basic Medical College, Changchun University of Traditional Chinese Medicine, Changchun 130117, China; gaoxu.1@icloud.com (X.G.); changyh2023@163.com (Y.C.); larissa07@163.com (Y.L.); 2 College of Food Science and Engineering, Jilin Agricultural University, Changchun 130118, China; tangping0220@163.com; 3 College of Traditional Chinese Medicine, Changchun University of Traditional Chinese Medicine, Changchun 130117, China; 25102701039@ccucm.edu.cn; 4 Nursing College, Changchun University of Traditional Chinese Medicine, Changchun 130117, China; shanealtus@163.com

**Keywords:** intracerebral hemorrhage, Nrf2, plant-derived natural compounds, ferroptosis, evidence grading

## Abstract

Intracerebral hemorrhage (ICH) is a devastating stroke subtype in which secondary brain injury is driven by oxidative stress, iron overload, ferroptosis, neuroinflammation, blood–brain barrier disruption, and defective hematoma clearance. Nrf2 is a redox-sensitive transcription factor that coordinates antioxidant defense, iron handling, inflammatory regulation, and neurovascular unit protection through downstream effectors such as HO-1, NQO1, GPX4, and SLC7A11. Plant-derived natural compounds have been widely investigated in experimental ICH models; however, increased Nrf2 expression or nuclear translocation alone does not establish Nrf2-dependent neuroprotection. Here, we critically appraise preclinical evidence linking plant-derived natural compounds to Nrf2-centered signaling in ICH and classify the evidence into three levels: causal Nrf2-dependent evidence, Nrf2-associated evidence, and indirect or context-transferred evidence. Representative flavonoids, phenolics, terpenoids, lignans, steroidal lactones, and other bioactive compounds are evaluated with attention to ICH-model relevance, causal pathway validation, pharmacokinetic limitations, brain exposure, and therapeutic window. Current evidence indicates that only a limited subset of compounds has been validated by genetic or pharmacological Nrf2 inhibition, whereas most remain supported by pathway association rather than causality, and preclinical studies rely predominantly on young healthy rodent models with early post-ICH intervention. Notably, no compound currently reaches relatively high translational priority because perihematomal brain exposure, delayed-treatment efficacy, and long-term safety evidence remain largely unavailable. We therefore propose an evidence-based prioritization framework integrating Nrf2 causality, ICH-specific efficacy, brain bioavailability, and translational readiness. This review clarifies the mechanistic boundaries of Nrf2-targeted natural compounds and outlines priorities for rigorous translational research in ICH.

## 1. Introduction

Intracerebral hemorrhage (ICH) is one of the most devastating subtypes of stroke and is associated with high mortality and long-term disability. Although advances in blood pressure control, anticoagulation reversal, minimally invasive hematoma evacuation, and stroke unit care have improved clinical management, effective pharmacological therapies for post-hemorrhagic secondary brain injury remain unavailable [1,2]. After hematoma formation, secondary brain injury evolves over hours to weeks and involves oxidative stress, iron dyshomeostasis, lipid peroxidation, ferroptosis, neuroinflammation, blood–brain barrier (BBB) disruption, cerebral edema, and neuronal death [3,4,5]. These processes are not isolated events but interact to form a self-amplifying injury network, making single-target intervention insufficient for the complex pathological progression of ICH.

Nuclear factor erythroid 2-related factor 2 (Nrf2) is a redox-sensitive transcription factor that regulates antioxidant defense, detoxification, iron handling, and cellular stress responses. Under basal conditions, Nrf2 is retained in the cytoplasm by Kelch-like ECH-associated protein 1 (Keap1) and undergoes proteasomal degradation. In response to oxidative or electrophilic stress, Nrf2 stabilizes, translocates into the nucleus, and binds antioxidant response elements (AREs), thereby inducing cytoprotective genes and proteins, including heme oxygenase-1 (HO-1), NAD(P)H quinone oxidoreductase 1 (NQO1), superoxide dismutase (SOD), glutamate–cysteine ligase catalytic subunit (GCLC), glutathione peroxidase 4 (GPX4), and solute carrier family 7 member 11 (SLC7A11; also known as xCT) [6,7]. In ICH, Nrf2 is not merely an antioxidant regulator; it also participates in ferroptosis suppression, inflammatory modulation, BBB protection, and hematoma resolution.

Experimental evidence indicates that Nrf2 activation may alleviate neurological deficits, brain edema, oxidative damage, and inflammatory responses after ICH, whereas Nrf2 deficiency impairs endogenous protective responses, including microglia/macrophage-mediated erythrophagocytosis and hematoma clearance [8,9]. However, Nrf2 signaling is highly context-dependent. In particular, HO-1, a major downstream target of Nrf2, may exert dual effects in hemorrhagic brain injury. Moderate and timely HO-1 induction may facilitate heme metabolism and antioxidant defense, whereas sustained or excessive HO-1 activation may increase free iron release and aggravate lipid peroxidation. Therefore, the therapeutic relevance of Nrf2 depends on the timing, magnitude, duration, and cellular context of pathway activation.

Plant-derived natural compounds, including flavonoids, phenolic compounds, terpenoids, lignans, and steroidal lactones, have attracted increasing attention as potential modulators of secondary brain injury after ICH. Representative compounds such as baicalin, baicalein, luteolin, resveratrol, curcumin, crocin, and kaempferol have been reported to attenuate oxidative stress, ferroptosis, neuroinflammation, BBB injury, and neurological dysfunction in experimental ICH models [10,11,12,13]. These compounds may engage multiple upstream pathways, including Keap1/Nrf2/ARE, PI3K/Akt/Nrf2, SIRT1/Nrf2, and p62/Keap1/Nrf2 signaling. Nevertheless, their translational limitations remain substantial, including poor aqueous solubility, limited metabolic stability, uncertain BBB penetration, inadequate brain exposure data, and insufficient long-term safety assessment.

Although studies on plant-derived natural compounds and Nrf2 signaling in ICH are rapidly increasing, the current evidence base remains uneven. A major limitation is that many studies infer Nrf2-dependent neuroprotection from increased Nrf2 nuclear translocation or upregulation of downstream proteins such as HO-1, NQO1, GPX4, or SLC7A11, without confirming whether the protective effects are abolished by Nrf2 knockout, small interfering RNA (siRNA)-mediated knockdown, selective pharmacological inhibition, or rescue experiments. Moreover, mechanistic findings from ischemic stroke, retinal degeneration, or other non-ICH models are sometimes extrapolated to ICH without sufficient consideration of hemorrhage-specific features, including hemoglobin toxicity, sustained iron overload, perihematomal inflammation, and hematoma clearance. These issues make it necessary to distinguish pathway association from pathway causality.

To address these gaps, this review does not aim merely to catalogue natural compounds that regulate Nrf2. Instead, we critically evaluate the strength of evidence linking plant-derived natural compounds to Nrf2-centered signaling in ICH. We first summarize ICH-specific Nrf2 regulation, with attention to temporal dynamics, cell-type specificity, and the dual role of HO-1. We then assess representative compounds using an evidence-grading framework that distinguishes causal Nrf2-dependent evidence, Nrf2-associated evidence, and indirect evidence derived from non-ICH contexts. Finally, we propose a compound-prioritization framework incorporating ICH model relevance, Nrf2 causal validation, brain exposure, pharmacokinetic feasibility, therapeutic window, safety, and long-term functional outcomes. By defining both the potential and the mechanistic boundaries of Nrf2-targeted natural compounds, this review aims to provide a more rigorous basis for future translational studies in ICH.

## 2. Literature Search Strategy and Evidence-Grading Framework

### 2.1. Literature Search Strategy

This review was designed as a structured narrative review with evidence grading rather than a formal systematic review or meta-analysis. Relevant studies were identified from PubMed, Web of Science, Embase, and CNKI through 4 June 2026. The search terms included combinations of the following keywords: “intracerebral hemorrhage”, “ICH”, “Nrf2”, “Keap1”, “HO-1”, “NQO1”, “GPX4”, “SLC7A11”, “ferroptosis”, “oxidative stress”, “neuroinflammation”, “blood–brain barrier”, “hematoma clearance”, “natural compounds”, “plant-derived compounds”, “flavonoids”, “phenolics”, “terpenoids”, “lignans”, “steroidal lactones”, “alkaloids”, and the names of individual compounds discussed in this review.

**Figure 1:**
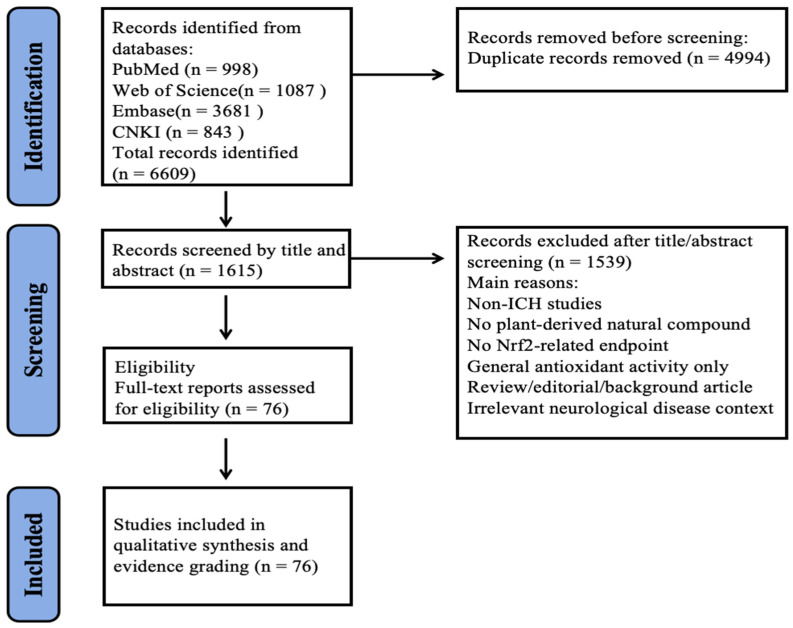
PRISMA-style flow diagram of literature identification, screening, eligibility assessment, and inclusion. Relevant studies were identified from PubMed, Web of Science, Embase, and CNKI through 4 June 2026. Records were screened according to predefined inclusion and exclusion criteria. Because this review was designed as a structured narrative review with evidence grading rather than a formal systematic review or meta-analysis, the PRISMA-style diagram was used to summarize the screening workflow and improve transparency rather than to claim full PRISMA-compliant systematic-review methodology.

The initial search identified 998 records from PubMed, 1087 from Web of Science, 3681 from Embase, and 843 from CNKI, yielding 6609 records in total. After removal of 4994 duplicates, 1615 records were screened by title and abstract, and 1539 were excluded because they were unrelated to ICH, did not involve plant-derived natural compounds, lacked Nrf2-related endpoints, described only general antioxidant activity, were review/editorial/background articles, or focused on irrelevant neurological disease contexts. Finally, 76 full-text reports were assessed for eligibility and included in the qualitative synthesis and evidence grading. Studies were included if they investigated plant-derived natural compounds in in vivo or in vitro ICH-related models and examined Nrf2 or Nrf2-related downstream molecules in the context of secondary brain injury; evidence from non-ICH models was considered only as indirect mechanistic support when clearly labeled. The literature identification and screening workflow is summarized in Figure 1. Because this review was designed as a structured narrative review with evidence grading rather than a formal systematic review or meta-analysis, the PRISMA-style flow diagram was used to improve transparency rather than to claim full PRISMA-compliant systematic-review methodology.

### 2.2. Evidence-Grading Criteria

To avoid overinterpreting pathway activation as pathway causality, the evidence for each compound was classified into three levels.

Level 1: Causal Nrf2-dependent evidence.

Compounds were classified as Level 1 when their protective effects in ICH-related models were validated by Nrf2 knockout, Nrf2 siRNA/shRNA, selective Nrf2 inhibitors such as ML385, or rescue experiments. In this category, attenuation or abolition of neuroprotection after Nrf2 blockade was considered essential evidence for Nrf2-dependent action.

Level 2: Nrf2-associated evidence.

Compounds were classified as Level 2 when they were tested in ICH-related models and shown to increase Nrf2 nuclear translocation or upregulate Nrf2 downstream targets, including HO-1, NQO1, GPX4, SLC7A11/xCT, SOD, or GCLC, but without genetic or pharmacological validation of Nrf2 dependence. These studies support an association with Nrf2 signaling but do not establish that Nrf2 is required for the observed neuroprotection.

Level 3: Indirect or context-transferred evidence.

Compounds were classified as Level 3 when their Nrf2-related mechanisms were mainly derived from non-ICH models, such as cerebral ischemia–reperfusion, traumatic brain injury, retinal degeneration, or other inflammatory and oxidative stress models. Such evidence may provide mechanistic hypotheses but should not be interpreted as direct support for ICH-specific Nrf2-dependent neuroprotection.

For borderline cases, partial attenuation by Nrf2 inhibition or knockout was considered Level 1 evidence only when the attenuation was statistically significant and occurred in at least one primary protective phenotype, such as neurological score, lesion volume, brain edema, hematoma volume, cell viability, or ferroptosis-related injury. When quantitative effect sizes were available, attenuation of ≥50% of the compound-induced improvement was interpreted as strong Nrf2-dependent evidence, whereas statistically significant but <50% attenuation was classified as partial Level 1 evidence and was downgraded in the translational prioritization score. If Nrf2 blockade altered only molecular markers but did not significantly reverse the protective phenotype, the evidence was classified as Level 2 rather than Level 1.

### 2.3. Translational Appraisal Criteria

For each compound, translational relevance was further evaluated according to the following criteria: ICH model relevance, timing of intervention, route of administration, dosing regimen, brain exposure or BBB penetration, pharmacokinetic limitations, safety and toxicity assessment, use of clinically relevant models, and long-term neurological outcomes. Particular attention was given to whether the studies used aged animals, female animals, hypertensive animals, comorbidity models, delayed treatment windows, and long-term functional endpoints.

Most available compound-level studies were performed in young healthy rodents and did not systematically evaluate sex-specific responses, aging, hypertension, anticoagulation status, diabetes, or other clinically relevant comorbidities. In the present prioritization framework, the absence of sex-specific analysis or clinically relevant age/comorbidity models was treated as a downgrading factor under therapeutic and model relevance. Therefore, compounds tested only in young healthy animals could not receive the highest translational/model-relevance score, even when their Nrf2-related molecular effects were positive.

To make the translational appraisal more operational, intervention timing was further categorized according to post-ICH treatment delay. Pretreatment or treatment initiated immediately after ICH was considered useful for mechanistic exploration but insufficient for high translational prioritization. Treatment initiated at ≥3 h after ICH was considered a minimally acceptable delayed-treatment window, whereas treatment initiated at ≥6–24 h after ICH was considered stronger evidence of translational feasibility. Long-term outcomes were handled similarly. Acute or subacute endpoints were sufficient for inclusion in the evidence assessment, but neurological or histological outcomes assessed at ≥28 days after ICH were considered necessary for high translational priority. Therefore, the absence of delayed-treatment testing, clinically relevant model validation, or ≥28-day outcomes did not exclude a compound from review, but it reduced its prioritization score.

This framework was used to distinguish compounds with stronger translational rationale from those supported mainly by preliminary, associative, or context-transferred evidence.

## 3. Nrf2 Signaling Pathway in Intracerebral Hemorrhage

### 3.1. Canonical Activation and Temporal Dynamics of Nrf2 After ICH

Nuclear factor erythroid 2-related factor 2 (Nrf2), a member of the CNC-basic leucine zipper (CNC-bZIP) transcription factor family, is widely recognized as a master regulator of cellular redox homeostasis, xenobiotic detoxification, and antioxidant defense [14,15]. Under physiological conditions, Nrf2 binds to Kelch-like ECH-associated protein 1 (Keap1), remains sequestered in the cytoplasm, and undergoes continuous degradation via the Cullin 3-dependent ubiquitin–proteasome pathway, thereby maintaining a low basal expression level [6,7]. This “inhibition–degradation” mechanism enables the rapid responsiveness of the Nrf2 pathway to oxidative or electrophilic stress.

Following ICH, hemoglobin, heme, and free iron released during hematoma degradation induce excessive production of reactive oxygen species (ROS), thereby creating a pronounced oxidative stress microenvironment. ROS and electrophilic molecules modify critical cysteine residues of Keap1 through oxidative reactions, leading to conformational changes that promote dissociation of Nrf2 from the Keap1 complex, prevent its degradation, and facilitate its nuclear translocation [16,17]. In the nucleus, Nrf2 forms heterodimers with small Maf proteins and binds to antioxidant response elements (AREs), initiating transcription of antioxidant and cytoprotective genes, including HO-1, NQO1, SOD, GCLC, GPX4, and SLC7A11 [7]. These downstream molecules collectively participate in ROS scavenging, glutathione metabolism, lipid peroxidation control, ferroptosis regulation, and iron homeostasis after ICH.

From a temporal perspective, Nrf2 signaling is rapidly activated after ICH and remains upregulated for several days. Experimental evidence indicates that Nrf2 nuclear translocation begins within hours after hemorrhage, peaks at approximately 8–24 h, and remains elevated thereafter. In human brain tissue, Nrf2 activation may persist for a longer period, suggesting that this pathway may contribute not only to acute injury containment but also to subacute and chronic repair processes [9,18]. Spatially, Nrf2 expression is concentrated mainly in mononuclear phagocytes within perihematomal regions, particularly microglia and infiltrating macrophages. These cells act as both mediators of inflammatory responses and key effectors of hematoma clearance and tissue repair.

Functional studies further support a central role for Nrf2 in the regulation of brain injury after ICH. Nrf2-knockout animals develop larger lesion volumes, more severe neurological deficits, increased oxidative stress, enhanced inflammatory responses, and aggravated mitochondrial and DNA damage. Conversely, Nrf2 activation may mitigate secondary brain injury through suppression of ROS accumulation, attenuation of lipid peroxidation, modulation of inflammatory responses, and enhancement of microglial phagocytosis [8,9]. Therefore, Nrf2 should not be viewed solely as an antioxidant regulator, but as an integrative signaling node linking oxidative stress, ferroptosis, neuroinflammation, blood–brain barrier injury, and hematoma resolution.

### 3.2. Cell-Type-Specific Nrf2 Signaling in ICH

The biological consequences of Nrf2 activation after ICH are highly dependent on cell type. Human perihematomal brain tissue has shown increased Nrf2 activation and upregulation of downstream transcripts such as HMOX1 and NQO1, supporting the clinical relevance of this pathway, although these observations do not establish cell-specific causality [18]. In neurons, Nrf2 activation is mainly associated with antioxidant defense, mitochondrial protection, and resistance to ferroptosis. By regulating downstream molecules such as SOD, NQO1, GCLC, GPX4, and SLC7A11, neuronal Nrf2 signaling may help preserve glutathione metabolism, reduce lipid ROS accumulation, and limit regulated cell death [7,19]. Experimental evidence also suggests that PPARγ may cooperate with Nrf2 to suppress neuronal ferroptosis after ICH, as Nrf2 inhibition with ML385 weakened the anti-ferroptotic effects of PPARγ activation [20]. However, neuronal Nrf2 activation alone is unlikely to fully explain post-ICH recovery, because inflammatory resolution, hematoma absorption, and neurovascular repair are largely mediated by non-neuronal cells.

In microglia and infiltrating macrophages, Nrf2 has a broader role that extends beyond antioxidant defense. Nrf2 activation may suppress excessive ROS production, limit NF-κB- and NLRP3-related inflammatory signaling, and promote a shift from inflammatory amplification toward clearance and repair [21,22,23,24,25]. Importantly, Nrf2 also enhances erythrophagocytosis and hematoma resolution. Previous studies have shown that Nrf2 activation promotes microglia/macrophage-mediated clearance of erythrocytes and hematoma components, whereas Nrf2 deficiency impairs CD36-related phagocytic activity and delays hematoma clearance [8,9]. This Nrf2–CD36-related clearance mechanism provides a direct link between oxidative-stress adaptation and microglia/macrophage-mediated hematoma removal [8]. Thus, in myeloid-lineage cells, Nrf2 may function as a regulator of both inflammatory tone and clearance capacity.

In astrocytes, Nrf2 signaling may contribute to antioxidant buffering, extracellular redox homeostasis, and support of the neurovascular unit. Astrocytes are closely associated with cerebral microvessels and participate in BBB maintenance. Nrf2 activation may reduce oxidative stress in the perihematomal region and preserve astrocyte-mediated vascular support. Moreover, Nrf2 induces tissue-specific upregulation of scavenger receptor A (SRA) in astrocytes, thereby enhancing astrocytic phagocytic capacity and accelerating hematoma resolution. Recent evidence further supports the Nrf2–SRA axis as a mechanism for glial-mediated hematoma clearance after ICH [26]. This suggests that astrocytes may actively participate in hematoma clearance, rather than serving only as supportive glial cells.

In brain endothelial cells and other components of the neurovascular unit, Nrf2 activation may protect BBB integrity by reducing oxidative stress, inhibiting matrix metalloproteinase activation, and preserving tight junction proteins such as ZO-1, occludin, and claudin-5 [19,27]. A recent ICH-focused study reported that Keap1/Nrf2 signaling was involved in Apelin-13-mediated preservation of tight junction proteins and reduction of BBB leakage, supporting the relevance of this axis to hemorrhage-induced barrier dysfunction [27]. Nevertheless, compared with microglia/macrophages, direct evidence for endothelial-cell-specific Nrf2 signaling in ICH remains relatively limited. Therefore, future studies should distinguish neuronal, microglial, astrocytic, macrophage, endothelial, and broader neurovascular-unit Nrf2 responses instead of interpreting whole-tissue Nrf2 upregulation as a uniform protective mechanism.

### 3.3. HO-1 as a Double-Edged Downstream Effector of Nrf2

Heme oxygenase-1 (HO-1) is one of the major downstream target genes of Nrf2 and is frequently used as an indicator of Nrf2 pathway activation. In the ICH microenvironment, hematoma degradation releases hemoglobin and heme, thereby promoting ROS generation, iron accumulation, lipid peroxidation, and ferroptosis. HO-1 catalyzes the degradation of free heme into biliverdin, carbon monoxide, and free iron; biliverdin is further converted to bilirubin, which has ROS-scavenging activity. Therefore, within this Nrf2-centered network, HO-1 represents a heme-metabolism node linking antioxidant defense, iron handling, and ferroptosis regulation, rather than a simple downstream protective marker [28].

The biological effect of HO-1 after ICH is highly dependent on timing, magnitude, and cellular context. Early or moderate HO-1 induction may facilitate heme detoxification, reduce heme-mediated oxidative injury, and support tissue repair. This protective effect is more likely to occur when HO-1 activation is coordinated with antioxidant and iron-buffering responses, including NQO1, GPX4, SLC7A11/xCT, ferritin, and glutathione metabolism [29]. However, HO-1 activation is not unconditionally protective in hemorrhagic brain injury. Because ICH involves sustained hemoglobin breakdown, heme accumulation, and iron overload, excessive or prolonged HO-1 expression may further increase the labile iron pool, thereby promoting Fenton chemistry, lipid peroxidation, and ferroptosis [30,31]. Consistent with this context-dependent interpretation, experimental evidence indicates that HO-1 exerts stage-dependent effects after ICH: excessive HO-1 activation in the early stage may aggravate brain edema, inflammation, oxidative stress, MMP-9/2 activation, and iron deposition, whereas longer-term HO-1 induction in the later stage may facilitate hematoma absorption, angiogenesis, and neurological recovery [32].

This dual role has important implications for evaluating Nrf2-targeted natural compounds. Increased HO-1 expression after compound treatment should not automatically be interpreted as evidence of Nrf2-dependent neuroprotection. Instead, HO-1 induction should be considered protective only when accompanied by reduced iron deposition, decreased lipid peroxidation, preserved GPX4/SLC7A11 activity, improved neurological outcomes, and, ideally, genetic or pharmacological validation using Nrf2 or HO-1 inhibition. Without such validation, HO-1 upregulation should be described as Nrf2-associated pathway activation rather than proof of Nrf2-dependent therapeutic action.

From a therapeutic perspective, the dual role of HO-1 also suggests that Nrf2-targeted compounds may need to be coordinated with iron-handling strategies rather than used as simple HO-1-amplifying interventions. Because HO-1-mediated heme degradation releases free iron, a rational approach would be to combine moderate Nrf2 activation with mechanisms that improve iron buffering, heme/hemoglobin scavenging, or iron clearance. Potential strategies include coordinated induction of ferritin and other iron-storage proteins, enhancement of haptoglobin–CD163- or hemopexin-mediated hemoglobin/heme handling, and carefully timed iron chelation. Such combinations may help reduce heme toxicity while limiting the labile iron pool that drives Fenton chemistry, lipid peroxidation, and ferroptosis. However, the timing and magnitude of these interventions are critical. Excessive early HO-1 activation or poorly timed iron chelation may theoretically interfere with hematoma resolution, erythrophagocytosis, or repair-associated iron recycling. Therefore, future studies evaluating Nrf2-targeted natural compounds should include iron-related endpoints, such as labile iron, ferritin heavy/light chains, ferroportin, hepcidin, haptoglobin, hemopexin, CD163, lipid peroxidation markers, hematoma volume, and long-term neurological outcomes.

**Figure 2:**
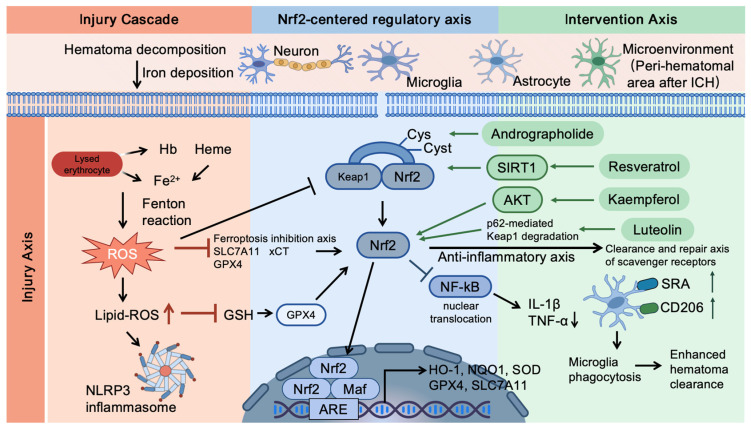
Proposed Nrf2-centered signaling network linking secondary brain injury and plant-derived natural compounds in ICH. Following ICH, hematoma degradation releases hemoglobin, heme, and free iron, thereby promoting reactive oxygen species (ROS) generation, iron deposition, lipid peroxidation, ferroptosis, neuroinflammation, blood–brain barrier (BBB) disruption, brain edema, and neurological dysfunction. Under oxidative or electrophilic stress, Nrf2 may dissociate from Keap1, escape ubiquitin–proteasome degradation, translocate into the nucleus, and bind antioxidant response elements (AREs), leading to the transcriptional activation of cytoprotective genes, including HO-1, NQO1, SOD, GCLC, GPX4, SLC7A11/xCT, and FTH1. Plant-derived natural compounds, including flavonoids, phenolic compounds, terpenoids, lignans, steroidal lactones, and other bioactive constituents, have been reported to modulate Nrf2-related pathways such as Keap1/Nrf2/ARE, PI3K/Akt/Nrf2, SIRT1/Nrf2, and p62/Keap1/Nrf2 signaling. These pathways may contribute to antioxidant defense, ferroptosis suppression, inflammasome inhibition, NF-κB-mediated inflammatory regulation, BBB preservation, and microglia/macrophage- or astrocyte-mediated hematoma clearance. However, the figure represents an evidence-informed mechanistic framework rather than definitive causal evidence for all compounds. The therapeutic relevance of Nrf2 activation depends on the timing, magnitude, duration, cellular context, and whether Nrf2 dependency has been validated by genetic or pharmacological approaches.

This position at the intersection of heme metabolism, oxidative stress, ferroptosis, and inflammation provides a conceptual rationale for evaluating Nrf2-centered signaling in ICH, while also requiring careful distinction between pathway association and causal pathway dependency (Figure 2).

## 4. Pharmacological Relevance of Nrf2 in Secondary Brain Injury After ICH

### 4.1. Oxidative Stress and Ferroptosis

Secondary brain injury after ICH represents a multidimensional pathological cascade triggered by the primary hemorrhagic event. This cascade involves oxidative stress, neuroinflammation, ferroptosis, and blood–brain barrier disruption and constitutes a major driver of neurological deterioration and poor prognosis [1,33]. Unlike the largely irreversible primary injury, secondary injury evolves in a time-dependent and therapeutically modifiable manner, making it a major target for intervention strategies [3]. Within this pathological context, Nrf2 acts as a redox-sensitive transcription factor positioned at the convergence of multiple injury pathways [7,9].

Under ICH-induced oxidative stress, stabilized Nrf2 translocates into the nucleus and binds antioxidant response elements (AREs), thereby inducing phase II detoxifying and antioxidant genes that form a multilayered cytoprotective network [19]. These Nrf2-regulated targets include NAD(P)H quinone oxidoreductase 1 (NQO1), superoxide dismutase (SOD), catalase (CAT), glutathione peroxidase 4 (GPX4), glutamate–cysteine ligase catalytic subunit (GCLC), glutathione reductase (GR), glutathione S-transferase (GST), thioredoxin, and thioredoxin reductase, which collectively stabilize intracellular redox homeostasis and the glutathione/glutathione disulfide (GSH/GSSG) balance [7,34]. These mechanisms are closely linked to ferroptosis inhibition, because GPX4 activity and SLC7A11/xCT-mediated cystine uptake are essential for limiting lipid peroxidation and iron-dependent cell death [7].

Pharmacological evidence further supports the antioxidant relevance of Nrf2 in ICH. The classical Nrf2 agonist sulforaphane promotes Nrf2 nuclear accumulation and upregulates antioxidant enzymes, including CAT, SOD, NQO1, and GST. These effects are accompanied by reduced oxidative damage markers such as 3-nitrotyrosine and 4-hydroxynonenal. Importantly, the neuroprotective effects of sulforaphane were abolished in Nrf2-deficient mice, supporting an Nrf2-dependent mechanism [34]. In addition, the novel Nrf2 activator RS9 enhances SOD expression, reduces ROS production, and alleviates brain edema during the subacute phase of ICH [35]. These findings support Nrf2 as a relevant target for oxidative stress and ferroptosis after ICH, while also illustrating that causal validation is required to define true Nrf2 dependency.

### 4.2. Hematoma Clearance

Accelerated hematoma clearance is considered an important therapeutic strategy for mitigating both primary and secondary brain injury after ICH. The timely removal of erythrocytes and their degradation products is central to this process. Nrf2 plays a key regulatory role in hematoma resolution by modulating the phagocytic capacity of brain-resident and infiltrating cells, particularly microglia, macrophages, and astrocytes.

Nrf2 has been shown to play a critical role in microglia-mediated erythrocyte clearance. In vitro, the Nrf2 agonist sulforaphane markedly enhances the phagocytic capacity of microglia toward erythrocytes and promotes Nrf2 nuclear translocation and DNA binding. These effects are accompanied by increased expression of downstream antioxidant enzymes, including GST, SOD1, and NQO1. In vivo, sulforaphane significantly reduces hematoma volume in wild-type mice, whereas this effect is abolished in Nrf2-knockout mice, together with delayed hematoma clearance. These findings indicate that sulforaphane-facilitated hematoma resolution is largely dependent on Nrf2 signaling [8].

Beyond microglia, Nrf2 also contributes to astrocyte-mediated hematoma clearance. Nrf2 induces tissue-specific upregulation of SRA in astrocytes, thereby enhancing astrocytic phagocytic capacity and accelerating hematoma resolution [26]. Evidence from other tissues also suggests that Nrf2 activators such as RS9 can enhance phagocytosis-related pathways, although these non-ICH findings should be interpreted only as indirect support rather than direct ICH-specific evidence [36,37]. Collectively, Nrf2 may serve as a molecular regulator of hematoma clearance by coordinating phagocytic responses in microglia/macrophages and astrocytes.

### 4.3. Neuroinflammation

The Nrf2 pathway serves as a key molecular switch in the regulation of secondary neuroinflammation after ICH. Hematoma degradation products, including hemoglobin, heme, and free iron, can function as damage-associated molecular patterns that activate microglia and infiltrating immune cells. This process triggers the TLR4/MyD88/NF-κB signaling cascade, leading to the release of pro-inflammatory cytokines such as IL-1β, TNF-α, and IL-6. Through multilayered crosstalk mechanisms, Nrf2 may antagonize NF-κB-mediated inflammatory signaling and attenuate inflammatory amplification after ICH [22].

Nrf2 and NF-κB signaling exhibit pronounced negative crosstalk. Nrf2 activation can indirectly attenuate ROS-driven NF-κB activation by enhancing antioxidant defenses and lowering intracellular ROS levels. Nrf2 may also stabilize IκB-α or reduce its proteasomal degradation, thereby restricting nuclear translocation of the NF-κB p65 subunit. In the nucleus, Nrf2 and NF-κB may further compete for the transcriptional coactivator CBP, limiting p65-mediated transcription of pro-inflammatory genes [22,23].

Beyond NF-κB signaling, Nrf2 also modulates NLRP3 inflammasome activation. After ICH, excessive ROS can serve as a danger signal for NLRP3 inflammasome priming and assembly, leading to caspase-1 activation and subsequent maturation and release of IL-1β and IL-18. By inducing antioxidant molecules such as HO-1, NQO1, SOD, CAT, and GPX4, Nrf2 limits ROS accumulation and thereby attenuates ROS-dependent NLRP3 activation. In parallel, Nrf2 may regulate the Trx1/TXNIP complex, reducing TXNIP binding to NLRP3 and suppressing inflammasome assembly [24].

p62/SQSTM1 serves as a key adaptor linking Nrf2-mediated anti-inflammatory signaling with inflammasome clearance. By binding to Keap1, p62 promotes Nrf2 stabilization and nuclear translocation, thereby amplifying Nrf2-dependent antioxidant and anti-inflammatory responses. Meanwhile, p62 recognizes ubiquitinated inflammasome components and delivers them to autophagosomes for degradation, establishing a negative feedback loop that limits excessive inflammasome-driven inflammation [24,25].

Pharmacological evidence further supports the involvement of Nrf2 in neuroinflammatory regulation after ICH. The Nrf2 activator omaveloxolone promotes Nrf2 nuclear accumulation and increases HO-1 and NQO1 expression. In OxyHb-stimulated microglia, omaveloxolone suppresses M1-like overactivation and ROS production while promoting an M2-like neuroprotective phenotype and improving mitochondrial function. Pretreatment with the selective Nrf2 inhibitor ML385 abolishes these protective effects, indicating that the anti-inflammatory and neuroprotective actions of omaveloxolone are largely dependent on Nrf2 signaling [21].

### 4.4. Blood–Brain Barrier Protection and Neurovascular Unit Homeostasis

Nrf2-mediated suppression of oxidative stress and neuroinflammation is closely interconnected with BBB protection. The BBB, a core structural component of the neurovascular unit, is composed of brain microvascular endothelial cells, tight junction proteins, basement membrane, pericytes, astrocytic endfeet, and microglia. After ICH, hematoma degradation products, ROS, inflammatory cytokines, and matrix metalloproteinase activation collectively disrupt tight junction proteins and basement membrane integrity, leading to increased BBB permeability, vasogenic brain edema, and inflammatory cell infiltration [19].

Nrf2 expression is predominantly localized to mononuclear phagocytes, including microglia and macrophages, while downstream Nrf2 target genes such as HMOX1 and NQO1 are upregulated in perihematomal tissues [18]. These observations suggest that Nrf2 may contribute to stabilization of the perihematomal microenvironment by regulating inflammatory cell behavior, redox homeostasis, and local repair responses. Nrf2 activation may directly or indirectly preserve BBB structural integrity by upregulating tight junction proteins, including ZO-1, occludin, and claudin-5, and by suppressing MMP activity [27].

Nrf2 may also attenuate neurovascular unit injury through anti-ferroptotic and anti-inflammatory mechanisms. Evidence from cerebral ischemia–reperfusion models indicates that Nrf2 activation can inhibit ACSL4 upregulation, restore GPX4 expression, reduce lipid peroxidation and inflammation, and improve BBB integrity [38]. Because these findings are derived from ischemic stroke rather than ICH models, they should be regarded as indirect mechanistic support requiring ICH-specific validation, rather than direct proof of ICH-specific BBB protection. In ICH, further studies are needed to clarify how endothelial, astrocytic, microglial, and pericytic Nrf2 signaling jointly regulate neurovascular repair.

Collectively, Nrf2 contributes to BBB protection and neurovascular unit homeostasis after ICH through suppression of oxidative stress and inflammation, regulation of the MMP/TIMP balance, preservation of tight junction proteins, and coordination of endothelial–astrocytic–microglial crosstalk [8,19,26,27]. These mechanisms further indicate that Nrf2 should be interpreted as a central signaling regulator linking inflammatory control, barrier repair, and tissue remodeling, rather than as a simple intracellular antioxidant factor.

### 4.5. Mechanistic Boundary: Nrf2 Association Versus Nrf2 Causality

Although the above evidence supports the biological relevance of Nrf2 in secondary brain injury after ICH, pathway activation should not be equated with therapeutic causality. Increased Nrf2 nuclear translocation or upregulation of HO-1, NQO1, GPX4, SLC7A11, or other downstream molecules indicates Nrf2-associated signaling, but does not by itself prove that Nrf2 is required for the observed neuroprotective phenotype. Causal evidence requires genetic or pharmacological validation, such as Nrf2 knockout, Nrf2 knockdown, selective Nrf2 inhibition, rescue experiments, or cell-type-specific pathway manipulation.

This distinction is particularly important for evaluating plant-derived natural compounds. Many available studies report Nrf2-related molecular changes after compound administration, but relatively few demonstrate that the protective effects are abolished when Nrf2 signaling is blocked. Therefore, in the following sections, compounds are not discussed simply as “Nrf2-dependent neuroprotective agents.” Instead, their evidence is classified according to whether the data support causal Nrf2-dependent protection, Nrf2-associated pathway activation, or indirect mechanistic relevance derived from non-ICH contexts.

## 5. Plant-Derived Natural Compounds Targeting Nrf2 in Experimental ICH

In recent years, accumulating preclinical evidence has suggested that various plant-derived natural compounds, including flavonoids, phenolics, terpenoids, lignans, steroidal lactones, and other bioactive constituents, may attenuate secondary brain injury in experimental intracerebral hemorrhage (ICH) models, with their effects frequently accompanied by modulation of Nrf2-related signaling. Nrf2 plays a central protective role in brain injury after ICH; accordingly, Nrf2-knockout mice exhibit larger lesion volumes, more severe neurological deficits, increased leukocyte infiltration, and enhanced reactive oxygen species (ROS) production following ICH [39,40]. These compounds are mainly derived from medicine–food homology plants and medicinal plants and have demonstrated multi-target pharmacological activities in preclinical studies [41]. 

Consistent with the mechanistic boundary defined above, the evidence levels and translational limitations of individual compounds are summarized in Table 3. To further operationalize translational appraisal, we constructed a compound-prioritization matrix that converts key limitations—including lack of brain concentration data, absence of delayed-treatment testing, use of young healthy animal models, and lack of long-term safety assessment—into explicit downgrading factors (Table 4). Importantly, Level 1 Nrf2 evidence was not considered equivalent to high translational priority, because causal pathway validation does not address brain exposure, delayed-treatment feasibility, model relevance, or long-term safety. The scoring criteria and priority interpretation are detailed in Table 4 notes.

### 5.1. Flavonoids

#### 5.1.1. Baicalein and Baicalin

Baicalein and its 7-O-glucuronide, baicalin, are representative bioactive flavonoids enriched in the roots of *Scutellaria baicalensis* Georgi, a plant belonging to the Lamiaceae family. They constitute an important material basis for the antiviral, antitumor, antibacterial, antioxidant, anti-inflammatory, hepatoprotective, and neuroprotective effects of this medicinal plant [42,43]. Baicalin is the glycosidic form of baicalein and can be hydrolyzed by β-glucuronidase to generate baicalein. In vivo, baicalein may further undergo phase II metabolism, including glucuronidation and sulfation. Therefore, although the pharmacological activities of baicalin and baicalein partially overlap, their mechanistic emphases are not completely identical [44].

In ICH-related studies, the effects of baicalin have mainly been associated with ferroptosis regulation. Using a collagenase-induced animal model of ICH and a hemin-induced in vitro model, Xie et al. found that baicalin activated Nrf2 signaling and upregulated the expression of HO-1, NQO1, SLC7A11/xCT, and GPX4, thereby reducing lipid ROS accumulation. These effects were accompanied by ferroptosis inhibition, improved cell viability, and amelioration of motor dysfunction and brain tissue injury, without obvious hepatic or renal toxicity [10]. Yu et al. further demonstrated that baicalin reduced iron deposition and hematoma volume and improved neurological function through the AKT/Nrf2/GPX4 axis. The protective effects of baicalin were attenuated by the AKT1 inhibitor LY294002 or an Nrf2 inhibitor, suggesting that AKT/Nrf2-related signaling may be involved in the anti-ferroptotic effects associated with baicalin treatment [45].

In contrast, baicalein appears to be more closely associated with antioxidant, anti-inflammatory, and blood–brain barrier (BBB)-protective mechanisms in ICH. Tang et al. reported that baicalein activated the Nrf2/ARE pathway through the miR-106a-5p/PHLPP2 axis, resulting in reduced neurological deficit scores, brain water content, and BBB permeability. Baicalein also decreased MDA levels and increased SOD and GSH-Px activities, thereby alleviating oxidative stress, neuronal apoptosis, and tissue injury [46]. In addition, baicalein may alleviate secondary brain injury after ICH by inhibiting ROS/NLRP3 inflammasome activation, regulating MMP expression, and attenuating BBB disruption [47,48].

Overall, baicalin appears to be more closely linked to Nrf2-related ferroptosis pathways, whereas baicalein is more closely associated with antioxidant, anti-inflammatory, and barrier-protective effects. However, the current evidence remains mainly derived from cellular and animal studies; therefore, their brain exposure, optimal dosing regimens, and clinical translational potential require further validation.

#### 5.1.2. Luteolin

Luteolin is a naturally occurring flavonoid compound that is widely present in *Reseda luteola* L. and exhibits various pharmacological activities, including antioxidant, anti-inflammatory, antitumor, antibacterial, and analgesic effects [49,50].

In ICH-related models, luteolin-induced protection has been associated with regulation of the p62/Keap1/Nrf2 signaling pathway. Studies have shown that luteolin enhances autophagy and promotes p62-mediated Keap1 degradation, thereby facilitating Nrf2 nuclear translocation and upregulating the expression of downstream antioxidant proteins, such as HO-1 and NQO1. These molecular changes are accompanied by suppression of oxidative stress markers, improved neurological scores, and attenuation of secondary brain injury indicators in experimental ICH models [51].

In addition, luteolin may exert cross-regulatory effects on inflammatory signaling. The Nrf2 downstream effectors HO-1 and NQO1 may indirectly suppress NF-κB-mediated inflammatory responses by reducing ROS-driven inflammatory activation [52]. Luteolin has also been reported to directly inhibit TLR4/TRAF6/NF-κB signaling in ICH models [53], though this effect appears to operate independently of Nrf2 and is therefore outside the primary mechanistic scope of this review. Nevertheless, direct validation that Nrf2 blockade abolishes luteolin-mediated protection remains absent, and the available evidence supports Nrf2-associated signaling rather than causal Nrf2-dependent neuroprotection.

#### 5.1.3. Epigallocatechin-3-Gallate

Epigallocatechin-3-gallate (EGCG) is a major catechin polyphenol enriched in tea, derived from *Camellia sinensis* (L.) Kuntze of the Theaceae family. It is also an important bioactive monomer responsible for the antioxidant, anti-inflammatory, and neuroprotective effects of green tea. By regulating oxidative stress, inflammatory responses, apoptosis, and multiple cytoprotective pathways, EGCG has shown potential protective effects in cardiometabolic diseases and neurological injury [54,55].

In rat models of ICH, EGCG promotes dissociation of the Keap1–Nrf2 complex, facilitates the translocation of Nrf2 from the cytoplasm to the nucleus, and regulates the expression of downstream ferroptosis-related molecules. Keap1 silencing further enhances the regulatory effects of EGCG on Nrf2-mediated ferroptosis-related mRNAs, whereas Nrf2 inhibition attenuates its anti-ferroptotic effects. These findings suggest that the Nrf2–Keap1 axis is involved in EGCG-associated attenuation of ferroptosis and brain injury after ICH [56].

Hao et al. further demonstrated, using ICH rats and in vitro neuronal models, that EGCG activates the Keap1/p62/Nrf2 signaling loop, increases nuclear Nrf2 expression, and upregulates key anti-ferroptotic proteins, including GPX4 and xCT. These effects are accompanied by inhibition of oxidative stress, iron deposition, and apoptosis. After intervention with the selective Nrf2 inhibitor ML385, the above protective effects were partially weakened, further supporting the involvement of Nrf2 in the antioxidant and anti-ferroptotic actions of EGCG [57]. In addition, EGCG may attenuate inflammation and microglial pyroptosis after ICH. Bao et al. found that EGCG pretreatment reduced the release of IL-1β, IL-18, and TNF-α, upregulated HO-1 expression, downregulated Caspase-1, GSDMD, and NLRP3 levels, and decreased the number of corresponding marker-positive microglia. EGCG also promoted microglial polarization from the M1 phenotype toward the M2 phenotype. These effects were partially reversed by the HO-1 inhibitor ZnPP, suggesting that EGCG may alleviate microglial pyroptosis and secondary inflammatory injury, at least in part, through the HO-1/Caspase-1/GSDMD/NLRP3 axis [58]. Despite these mechanistic findings, EGCG still faces pharmacokinetic limitations, including limited stability, modest bioavailability, and uncertain effective brain exposure.

#### 5.1.4. Epicatechin

(-)-Epicatechin (EC) is a naturally occurring flavanol polyphenol that is widely distributed in tea from *Camellia sinensis* and cocoa from *Theobroma cacao*, and has been reported to exert antioxidant and anticancer effects [59,60].

In ICH, EC can promote nuclear accumulation of Nrf2 in astrocytes and upregulate the expression of antioxidant proteins such as SOD1. Even under Nrf2-knockout conditions, EC can still downregulate HO-1 expression by inhibiting the AP-1 pathway. Given that excessive HO-1 activation after ICH can promote heme degradation and free iron release, thereby inducing iron-dependent lipid peroxidation, EC may help reduce cerebral iron deposition and alleviate secondary injury by regulating the AP-1/HO-1 axis. In addition, this pathway may reduce MMP-9 activity and Lcn2 levels, while suppressing the expression of ferroptosis-related genes [61,62]. Therefore, EC should be interpreted as a compound involving both Nrf2-associated antioxidant responses and Nrf2-independent AP-1/HO-1 regulation.

#### 5.1.5. Isoliquiritigenin

Isoliquiritigenin (ILG) is a naturally occurring chalcone compound mainly derived from the dried roots and rhizomes of plants of the genus *Glycyrrhiza* L. in the family Fabaceae. ILG has been reported to exert a wide range of pharmacological activities, including anti-inflammatory, anti-influenza, anti-tyrosinase, antibacterial, anti-sarcoma, antioxidant, anti-leiomyoma, anti-cholera, anti-asthmatic, antidiabetic, and anticancer effects, as well as neuroprotective, hepatoprotective, and cardioprotective actions [63,64].

In experimental models of ICH, ILG has been associated with protection through the Nrf2–ROS/NF-κB–NLRP3 axis. ILG treatment activates the Nrf2-mediated endogenous antioxidant system, suppresses ROS generation and NF-κB p65 activation, and subsequently blocks the assembly and activation of the NLRP3 inflammasome, thereby attenuating blood–brain barrier disruption, brain edema, and neuronal degeneration. Nrf2 gene silencing not only aggravates neurological deficits and inflammatory responses after ICH, but also abolishes the protective effects of ILG, indicating that Nrf2 signaling is functionally required for its protective effects in the cited model [65]. Further studies are still needed to define its pharmacokinetic properties, brain exposure, dose–response relationship, therapeutic window, and long-term safety.

#### 5.1.6. Kaempferol

Kaempferol is a naturally occurring flavonol compound widely found in tea plants (*Camellia sinensis*), broccoli, and various fruits and medicinal plants. It has been reported to possess antioxidant, anti-inflammatory, antibacterial, anticancer, cardioprotective, neuroprotective, antidiabetic, anti-osteoporotic, estrogenic/anti-estrogenic, anxiolytic, analgesic, and anti-allergic activities [66].

In ICH models, kaempferol increases the p-AKT/AKT ratio, promotes AKT phosphorylation and activation, facilitates Nrf2 nuclear translocation, and enhances HO-1 expression. These molecular changes are accompanied by reduced oxidative stress markers, decreased apoptotic indices, and improved neurological scores in experimental models [12]. However, whether Nrf2 is required for kaempferol-mediated protection remains to be confirmed by Nrf2 knockdown, selective inhibition, or rescue experiments.

#### 5.1.7. Calycosin

Calycosin is a naturally occurring isoflavone bioactive compound mainly derived from *Astragalus membranaceus*. Modern pharmacological studies have shown that calycosin exhibits a broad spectrum of biological activities, including anticancer, anti-inflammatory, anti-osteoporotic, neuroprotective, and hepatoprotective effects. Accordingly, it has attracted sustained attention in the fields of cardiovascular and cerebrovascular diseases, neurological disorders, and cancer [67,68,69].

In an experimental ICH model, calycosin treatment at 50 mg/kg alleviated neurological deficits and brain edema and was superior to vehicle treatment in reducing lesion volume, blood accumulation, and hemispheric swelling. Mechanistically, these effects are accompanied by changes consistent with Nrf2-related antioxidant pathway activation, reduced oxidative stress markers, and attenuation of NF-κB- and NLRP3-associated inflammatory responses [70]. However, direct validation that Nrf2 blockade abolishes calycosin-mediated protection remains insufficient.

Overall, flavonoids show heterogeneous levels of Nrf2-related evidence in ICH models. ILG and EGCG have relatively stronger causal or pharmacological validation, whereas luteolin, kaempferol, calycosin, baicalein, and several baicalin-related findings are mainly supported by Nrf2-associated molecular changes. Detailed evidence levels, validation approaches, and translational limitations are summarized in Table 3.

### 5.2. Phenolic Compounds

#### 5.2.1. Resveratrol

Resveratrol is a naturally occurring multitarget non-flavonoid polyphenolic compound belonging to the stilbene class. It is mainly found in plant sources such as grapes, peanuts, and cocoa, and has been widely investigated because of its antioxidant, anti-inflammatory, cardioprotective, hepatoprotective, neuroprotective, immunomodulatory, and anticancer properties [71,72].

In a collagenase-induced mouse model of ICH, intravenous administration of resveratrol (10 mg/kg) as late as 30 min after ICH significantly improved acute neurological deficits, alleviated brain edema, promoted hematoma resolution, and decreased the expression of pro-inflammatory cytokines such as IL-1β [73]. These findings support the ICH-related pharmacological relevance of resveratrol.

Resveratrol may be associated with activation of the SIRT1/Nrf2 signaling cascade. It promotes activation of the Nrf2-ARE pathway through SIRT1-mediated deacetylation and upregulates the expression of antioxidant enzymes such as HO-1 and NQO1. Studies using ischemia/reperfusion models further indicate that resveratrol can suppress oxidative stress through the Nrf2/Shh signaling axis and induce microglial polarization from the M1 phenotype toward the M2 phenotype [74]. In non-ICH cerebrovascular disease models, the observed effects of resveratrol are markedly attenuated or abolished following treatment with Nrf2 inhibitors, such as ML385, or in Nrf2-/- knockout mice [75]. However, because this mechanistic evidence is derived entirely from non-ICH models, it constitutes indirect support rather than ICH-specific causal validation.

#### 5.2.2. Gastrodin

Gastrodin is a naturally occurring phenolic glycoside and one of the major bioactive constituents of the dried tubers of *Gastrodia elata* Blume, a plant belonging to the Orchidaceae family. Modern pharmacological studies have shown that gastrodin exhibits a broad range of biological activities, particularly pronounced neuroprotective, antioxidant, anti-inflammatory, and anti-apoptotic effects in central nervous system disorders [76,77].

Gastrodin attenuates oxidative stress and neuronal apoptosis after experimental ICH, and these effects are associated with modulation of the Keap1/Nrf2/HO-1 signaling axis. Studies have shown that gastrodin increases SOD and GSH-Px activities and reduces the levels of ROS, MDA, 8-OHdG, and 3-NT, thereby alleviating oxidative damage to lipids, DNA, and proteins. Meanwhile, gastrodin upregulates Bcl-2 expression and downregulates Bax and cleaved caspase-3/9 levels, ultimately reducing apoptotic indices and improving neurological scores in experimental ICH models [78]. These findings are consistent with Nrf2/HO-1-associated antioxidant protection, but direct Nrf2-dependency validation remains insufficient.

#### 5.2.3. Curcumin

Curcumin is the principal polyphenolic bioactive constituent of the dried rhizomes of *Curcuma longa* L., a plant belonging to the Zingiberaceae family. It has been reported to possess antioxidant, anti-inflammatory, antimutagenic, antibacterial, and anticancer activities [79,80].

Curcumin has been shown to suppress heme-induced oxidative stress, reduce ROS and MDA levels, and upregulate the expression of Nrf2 and its downstream target genes, including HO-1, NQO1, and GPX4. These effects are accompanied by prevention of endogenous GSH depletion and neurological improvement [81]. To overcome the poor water solubility and limited in vivo stability of curcumin, platelet membrane-coated curcumin-loaded PLGA nanoparticles (PCNPs) have been developed to enhance targeted delivery to ICH lesions. PCNPs were associated with suppression of neuroinflammation and excessive astrocyte proliferation, astrocyte-to-neuron transdifferentiation, and improved tissue outcomes in experimental settings [82]. Nevertheless, direct validation that Nrf2 blockade abolishes curcumin-mediated protection remains limited, and poor aqueous solubility, low systemic bioavailability, rapid metabolism, and uncertain brain exposure remain major translational concerns.

#### 5.2.4. Protocatechuic Acid

Protocatechuic acid (PCA) is a naturally occurring phenolic acid widely found in *Salvia miltiorrhiza* Bunge of the Lamiaceae family, *Oryza sativa* L., and *Allium cepa* L. PCA exhibits a wide range of biological activities, including antioxidant, antibacterial, anticancer, anti-ulcer, antidiabetic, anti-aging, antifibrotic, antiviral, anti-inflammatory, analgesic, anti-atherosclerotic, cardioprotective, hepatoprotective, neuroprotective, and renoprotective effects [83,84].

Studies have shown that intraperitoneal administration of PCA at 30 mg/kg significantly reduces brain water content in the ipsilateral hemorrhagic hemisphere on day 3 after ICH. Mechanistically, this effect is associated with the promotion of Nrf2 nuclear translocation and upregulation of HO-1 expression. PCA also downregulates AQP4 expression and increases the levels of tight junction proteins, such as ZO-1 and occludin, thereby alleviating brain edema and improving blood–brain barrier integrity. After Nrf2 knockdown, the PCA-induced upregulation of HO-1, ZO-1, and occludin is markedly attenuated, suggesting that Nrf2 signaling contributes functionally to PCA-mediated barrier protection. Furthermore, PCA may protect tight junctions in brain microvascular endothelial cells by inhibiting the ROS–MMP cascade, while also suppressing AQP4 expression in astrocytes. In this way, PCA may exert synergistic protective effects on the neurovascular unit [85].

#### 5.2.5. Pterostilbene

Pterostilbene (PTE) is a naturally occurring stilbene polyphenol and a dimethylated derivative of resveratrol. It is mainly found in plants of the genus *Vaccinium* spp. In the Ericaceae family and *Pterocarpus* spp. in the Fabaceae family. PTE has been reported to exert protective effects against oxidative injury, neuroinflammation, apoptosis, cholinesterase activity, and cancer [86,87].

In models of cerebral hemorrhage, PTE has been associated with protection through the SIRT1/Nrf2 axis. In vivo and in vitro experiments have shown that PTE activates SIRT1-mediated Nrf2 signaling, accompanied by suppression of ICH-induced oxidative injury and neuronal apoptosis. Reverse validation experiments showed that when SIRT1 was specifically inhibited, oxidative stress and apoptosis remained elevated despite PTE treatment, supporting the involvement of SIRT1-mediated signaling in the antioxidant network activated by PTE [88].

In addition, PTE has been shown to remodel mitochondrial dynamics in microglia via OPA1-dependent mitochondrial fusion, reducing microglial inflammation, blood–brain barrier injury, and brain edema; OPA1 conditional knockout largely abolishes these benefits [89]. These findings indicate that PTE acts through both Nrf2-associated and Nrf2-independent mitochondrial mechanisms, the latter of which falls outside the primary mechanistic scope of this review. No study has employed Nrf2 knockout or selective inhibition in ICH models to confirm Nrf2 dependency for PTE’s protective effects.

#### 5.2.6. Salvianolic Acid A

Salvianolic acid A (SAA) is one of the major water-soluble polyphenolic constituents of *Salvia miltiorrhiza* Bunge, a plant of the genus Salvia in the Lamiaceae family. It has been reported to exhibit antioxidant, anti-inflammatory, anticancer, neuroprotective, and antifibrotic activities [90,91].

In experimental ICH models, SAA promotes nuclear accumulation of Nrf2 and enhances its transcriptional activity, upregulates the expression of anti-ferroptotic proteins such as GPX4 and xCT, reduces MDA, ACSL4, and Fe^2+^ levels in perihematomal tissues, and alleviates glutathione depletion, thereby inhibiting ferroptosis. Both the AKT inhibitor SH-6 and the Nrf2 inhibitor ML385 reverse these protective effects, suggesting that AKT/Nrf2-related signaling is functionally involved in SAA-mediated anti-ferroptotic protection [90]. Other studies have shown that, in brain microvascular endothelial cell models, SAA can also alleviate oxidative stress and mitochondrial dysfunction through Nrf2-mediated antioxidant mechanisms. Although some evidence derives from studies of ischemic stroke, these findings may provide indirect mechanistic reference for understanding how SAA may maintain neurovascular unit homeostasis [92].

#### 5.2.7. Polydatin

Polydatin is a naturally occurring resveratrol glycoside mainly derived from *Polygonum cuspidatum*, a plant of the Polygonaceae family. It has been reported to exert antiviral, antibacterial, anti-inflammatory, neuroprotective, and cardioprotective effects [93].

In ICH models, polydatin significantly reduces modified neurological severity scores (mNSS), brain water content, and serum NSE levels, suggesting a neuroprotective effect. Mechanistically, polydatin alleviates oxidative stress-induced injury by reducing NO and MDA levels and increasing SOD, GSH, and GSSG levels. It also upregulates the expression of Nrf2 and its downstream targets NQO1 and HO-1, suggesting that polydatin-associated effects are accompanied by activation of Nrf2-ARE-related signaling [94]. However, direct Nrf2-dependency validation using knockout, knockdown, selective inhibition, or rescue approaches remains insufficient.

Overall, phenolic compounds show diverse levels of evidence. PCA and SAA have relatively stronger pathway-validation evidence, whereas gastrodin, curcumin, PTE, and polydatin are mainly supported by Nrf2-associated molecular changes. Resveratrol has ICH efficacy evidence, but part of its Nrf2-causality evidence is transferred from non-ICH models. Detailed evidence levels and translational appraisal are summarized in Table 3.

### 5.3. Terpenoids

#### 5.3.1. Andrographolide

Andrographolide is a naturally occurring diterpenoid lactone mainly extracted from the dried aerial parts of *Andrographis paniculata* (Burm. f.) Nees, a plant of the genus Andrographis in the Acanthaceae family. It has been reported to exert anti-inflammatory, antioxidant, anticancer, antibacterial, and antihyperglycemic effects [95,96].

Andrographolide can activate the Nrf2/HO-1 signaling pathway by modifying key cysteine residues in Keap1, thereby inhibiting Nrf2 ubiquitination and degradation and promoting its nuclear translocation. This process enhances the activities of endogenous antioxidant enzymes, such as SOD and CAT, reduces MDA levels, and attenuates ICH-induced disruption of the blood–brain barrier and blood–cerebrospinal fluid barrier by inhibiting NF-κB-mediated inflammatory responses and downregulating MMP-9 expression, thereby attenuating secondary brain injury indicators in the cited experimental model [97]. However, whether Nrf2 activation is required for these protective effects remains to be verified using direct pathway-blockade approaches.

#### 5.3.2. Crocin

Crocin is a water-soluble apocarotenoid, broadly classified as a tetraterpenoid derivative, that is widely present in the stigmas of *Crocus sativus* L., a plant of the genus Crocus in the Iridaceae family, and in the fruits of *Gardenia jasminoides* Ellis of the Rubiaceae family. It has been reported to exhibit antioxidant, anti-inflammatory, anti-apoptotic, lipid-regulating, memory-improving, and tumor growth-inhibitory effects [98,99,100].

In models of intracerebral hemorrhage, crocin significantly increases the activities of SOD and GSH-Px in perihematomal tissues and reduces the level of the lipid peroxidation product MDA. It also inhibits neuronal ferroptosis, as evidenced by reduced local Fe^2+^ concentrations and upregulation of ferroptosis-regulatory proteins, including GPX4, FTH1, and SLC7A11. These effects are accompanied by increased Nrf2 expression and nuclear translocation, representing Nrf2-associated rather than causally validated pathway activation [101]. However, direct Nrf2-dependency validation has not been clearly demonstrated in the cited study.

Overall, the terpenoid-related compounds included in this review are mechanistically relevant to Nrf2-associated antioxidant and anti-ferroptotic signaling. However, andrographolide and crocin require direct pathway validation before being regarded as Nrf2-dependent neuroprotective candidates in ICH. Their evidence levels and translational limitations are summarized in Table 3.

### 5.4. Other Natural Compounds

#### 5.4.1. Phillyrin

Phillyrin is a lignan glycoside and one of the major bioactive constituents of *Forsythia suspensa* (Thunb.) Vahl, a plant of the genus Forsythia in the Oleaceae family. It is widely distributed in the fruits, leaves, and stems of this plant, with the dried fruit, known as Forsythiae Fructus, serving as the traditional medicinal source. Phillyrin exhibits multiple pharmacological activities, including anti-inflammatory, anti-obesity, antitumor, antibacterial, and antiviral effects [102,103].

Phillyrin significantly alleviates neurological deficits and reduces intracerebral lesion volume in animal models of ICH, while also attenuating neuronal apoptosis in gray matter and white matter injury around the lesion. Mechanistic studies have shown that Phillyrin activates the Nrf2 signaling pathway and promotes Nrf2 nuclear translocation, thereby upregulating the expression of antioxidant-related molecules, including HO-1, NQO1, and SOD-1. These effects reduce ROS accumulation and MDA levels, ultimately attenuating oxidative stress-induced injury. The Nrf2-specific inhibitor ML385 reverses the antioxidant and anti-apoptotic effects of phillyrin, suggesting functional involvement of Nrf2 signaling in phillyrin-mediated neuroprotection [104].

#### 5.4.2. Withaferin A

Withaferin A (WFA) is a representative steroidal lactone compound, also known as a withanolide. It is mainly derived from *Withania somnifera* (L.) Dunal, a plant of the Solanaceae family, and is distributed in tissues such as the leaves and roots. WFA possesses anti-inflammatory, antioxidant, and anticancer activities, and also exhibits marked anti-invasive and anti-angiogenic effects [105,106].

In a hemin-induced SH-SY5Y neuronal model of ICH-related injury, WFA promoted Nrf2 nuclear translocation, upregulated HO-1, increased GPX4 and GSH levels, and reduced Fe^2+^ accumulation and ROS production. These effects were associated with ferroptosis inhibition and were further enhanced when WFA was combined with Ferrostatin-1. Importantly, either Nrf2 silencing or HO-1 inhibition reversed WFA-mediated HO-1 upregulation and neuroprotection, indicating functional involvement of the Nrf2/HO-1 cascade in this in vitro model [107]. However, this evidence is mainly derived from an in vitro hemin-induced neuronal model and requires in vivo validation.

#### 5.4.3. Betaine

Betaine (BET), also known as glycine betaine or N, N, N-trimethylglycine, is a naturally occurring zwitterionic quaternary ammonium compound. It functions as an osmoprotectant and methyl donor, participating in the maintenance of cellular homeostasis and the regulation of one-carbon metabolism. BET is widely distributed in plants, animals, and microorganisms. Typical dietary and botanical sources of betaine include *Beta vulgaris* L. (beetroot or sugar beet), *Spinacia oleracea* L. (spinach), and *Lycium barbarum* L. (goji berry). BET has been reported to exert anti-inflammatory, antioxidant, anti-aging, anticancer, and multiorgan-protective effects, and may improve vascular risk factors [108,109,110].

BET effectively promotes local hematoma resolution and significantly reduces abnormal iron deposition in brain tissue. It markedly decreases ROS production, the lipid peroxidation product MDA, and the level of ACSL4, a key pro-ferroptotic enzyme, while significantly increasing the expression of the endogenous antioxidant GSH and GPX4, a core ferroptosis-defense enzyme. Inhibition of Nrf2 substantially weakens the neuroprotective effects of BET, including its anti-edematous, antioxidant, and anti-ferroptotic actions, supporting functional involvement of the Nrf2/HO-1 pathway in the cited model [111]. Whether BET reaches effective concentrations in perihematomal brain tissue and whether its methyl-donor properties influence long-term outcomes require further investigation.

#### 5.4.4. Silymarin

Silymarin (SIL) is a naturally occurring mixture of flavonolignan bioactive compounds derived from *Silybum marianum*, a medicinal plant belonging to the Asteraceae family. Unlike single-molecule natural compounds, SIL is composed of several structurally related flavonolignans, mainly including silybin, isosilybin, silychristin, and silydianin. SIL has been reported to exhibit antioxidant, anti-apoptotic, anti-inflammatory, hepatoprotective, neurotrophic, estrogen-like, and endocrine-modulatory activities [112,113,114].

Studies have shown that oxidative stress is significantly elevated 24 h after ICH, as reflected by increased ROS production and lipid peroxidation. Administration of silymarin at 200 mg/kg 30 min after injury reduced oxidative damage and enhanced antioxidant capacity. Meanwhile, silymarin inhibits NF-κB p65 activation, downregulates the NLRP3 inflammasome and its downstream effectors caspase-1 and IL-1β, and upregulates the Nrf2/HO-1 signaling pathway, thereby reducing inflammatory responses and protecting against secondary brain injury after ICH [115]. However, direct genetic or pharmacological validation of Nrf2 dependency has not been clearly demonstrated in the cited study.

#### 5.4.5. Sulforaphane

Sulforaphane (SFN) is a naturally occurring isothiocyanate derived from glucosinolate precursors in cruciferous vegetables, particularly broccoli and other Brassicaceae plants. Unlike many polyphenolic compounds that are mainly discussed as antioxidant or anti-inflammatory agents, SFN is widely used as a representative plant-derived activator of the Keap1/Nrf2/ARE pathway. It has been reported to exhibit antioxidant, anti-inflammatory, cytoprotective, detoxifying, and neuroprotective activities in experimental models of neurological injury [34,116].

In experimental ICH, SFN promotes Nrf2 nuclear accumulation and upregulates Nrf2-regulated antioxidant enzymes, including catalase, superoxide dismutase, NAD(P)H quinone oxidoreductase 1, and glutathione S-transferase. These molecular changes are accompanied by reduced oxidative damage and improved neurological outcomes after ICH. Importantly, the protective effects of SFN were abolished in Nrf2-deficient mice, indicating that its antioxidant and neuroprotective effects are strongly dependent on Nrf2 signaling [34]. Beyond acute antioxidant defense, SFN enhanced microglia-mediated erythrocyte phagocytosis and accelerated hematoma clearance. This effect was associated with Nrf2 nuclear translocation, increased expression of downstream antioxidant molecules, and reduced hematoma volume in wild-type mice, whereas the hematoma-clearance effect was lost in Nrf2-knockout mice [8].

Overall, compounds in this category show variable evidence strength. Phillyrin, WFA, BET, and SFN have relatively stronger causal or pharmacological support, whereas silymarin is mainly supported by Nrf2-associated molecular changes and phenotypic improvement. This section summarizes recent advances in representative classes of these compounds, with Table 1. Providing a concise overview of compound classes and sources, and Table 2 summarizing their Nrf2-related regulatory mechanisms and pharmacological effects in ICH models. Detailed evidence levels, causal validation approaches, and translational limitations are summarized in Table 3.

**Table 1:** Summary of representative plant-derived natural compounds discussed in this review.

Class	Representative Compounds	Main Natural Sources	Main Reported Bioactivities	Refs.
Flavonoids	Baicalein, Baicalin, Luteolin, EGCG, EC, ILG, Kaempferol, Calycosin	*Scutellaria baicalensis*, tea, cocoa, *Glycyrrhiza* spp., *Astragalus membranaceus*, fruits and vegetables	Antioxidant, anti-inflammatory, anti-ferroptotic, neuroprotective	[42,43,44,45,46,47,48,49,50,51,52,53,54,55,56,57,58,59,60,61,62,63,64,65,66,67,68,69,70]
Phenolics	Resveratrol, Gastrodin, Curcumin, PCA, PTE, SAA, Polydatin	Grapes, peanuts, *Gastrodia elata*, *Curcuma longa*, *Salvia miltiorrhiza*, *Polygonum cuspidatum*	Antioxidant, anti-inflammatory, anti-apoptotic, neuroprotective, vascular-protective	[71,72,73,74,75,76,77,78,79,80,81,82,83,84,85,86,87,88,89,90,91,92,93,94]
Terpenoids	Andrographolide, Crocin	*Andrographis paniculata*, *Crocus sativus*, *Gardenia jasminoides*	Antioxidant, anti-inflammatory, anti-apoptotic, anti-ferroptotic	[95,96,97,98,99,100,101]
Other categories	Phillyrin, Withaferin A, Betaine, Silymarin, Sulforaphane	*Forsythia suspensa*, *Withania somnifera*, *Beta vulgaris*, *Silybum marianum*, cruciferous vegetables	Antioxidant, anti-inflammatory, neuroprotective, metabolism-related protective effects	[102,103,104,105,106,107,108,109,110,111,112,113,114,115,116]

**Table 2:** Nrf2-related pharmacological effects of plant-derived natural compounds in ICH-related experimental models.

Class	Active Ingredients	Experimental Model	Impact on	Refs.
Flavonoids	Baicalin	Male C57BL/6 mice injected with type IV collagenase	↑: HO-1,NQO1,SLC7A11 (xCT),GPX4 ↓: ROS	[10]
		Hemin-induced mouse HT22 hippocampal neuronal cells	↑: SLC7A11 (xCT), GPX4, GSH ↓: ROS, Fe^2+^	
		Male SD rats that received autologous blood injection	↑: Nrf2, HO-1, GPX4 ↓: MDA	[45]
	Baicalein	Male SD rats injected with type IV collagenase	↑: SOD, GSH-Px, Nrf2/ARE ↓: MDA	[46]
		Male C57BL/6 mice injected with collagenase; primary cortical neurons from mice treated with oxyhemoglobin	↑: Nrf2 ↓: ROS, NLRP3, Caspase-1 p20, IL-1β, GSDMD-N	[47,48]
	Luteolin	Male SD rats that received autologous blood injection	↑: p62, Nrf2, HO-1, NQO1 ↓: Keap1, ROS, MDA, TLR4, TRAF6, NF-κB p65 phosphorylation, TNF-α, IL-1β, IL-6	[51,52,53]
	EGCG	Male SD rats that received autologous blood injection	↑: Nrf2, GPX4, xCT (SLC7A11), HO-1 ↓: ROS, TNF-α, IL-1β	[56]
		Hemoglobin-treated primary cortical neurons from neonatal SD rats	↑: GPX4, Nrf2, HO-1 ↓: Caspase-1, GSDMD, NLRP3, IL-1β, IL-18	[57]
	EC	Hemoglobin-treated primary cortical astrocytes from neonatal CD-1 mice (P1–P2)	↑: Nrf2, SOD1 ↓: HO-1, MMP-9, Lcn2	[61]
		Male CD-1 mice injected with collagenase	↑: Nrf2, SOD1	
	ILG	Male C57BL/6 mice that received collagenase injection	↑: Nrf2, HO-1, NQO1 ↓: ROS, NF-κB p65,NLRP3,Caspase-1,IL-1β	[65]
	Kaempferol	Male SD rats that received autologous blood injection	↑: p-AKT/AKT ratio, Nrf2, HO-1 ↓: ROS, MDA, TUNEL-positive cells	[12]
		Rat PC12 pheochromocytoma cell line	↑: p-AKT, Nrf2, HO-1 ↓: ROS	
	Calycosin	Male ICR mice that received collagenase injection	↑: Nrf2 ↓: NF-κB p65,NLRP3, Caspase-1, IL-1β, IL-18, MDA, ROS	[70]
Phenolics	Resveratrol	OGD/R-treated microglial model (non-ICH mechanistic evidence)	↑: SIRT1, Nrf2, HO-1	[74]
		Male SD rats that received autologous blood injection	↑: Nrf2,HO-1, NQO1, SOD, GSH ↓: IL-1β, TNF-α, MDA	[73]
	Gastrodin	Male SD rats that received autologous blood injection	↑: SOD, GSH-Px, Bcl-2, Nrf2, HO-1 ↓: ROS, MDA, 8-OHdG, 3-NT, Bax, Caspase-3, Caspase-9, TUNEL	[78]
	Curcumin/Curcumin-loaded nanoparticles	Primary cortical neurons from neonatal SD rats	↑: Nrf2, HO-1, NQO1 ↓: ROS, MDA	[81]
		Male SD rats that received autologous blood injection	↑: Nrf2, HO-1, NQO1, GPX4, GSH ↓: MDA	
		Male SD rats injected with type IV collagenase	↑: Nrf2, GPX4, GSH ↓: ROS	[82]
	PCA	Male C57BL/6 mice that received collagenase injection	↑: Nrf2, HO-1, ZO-1, Occludin ↓: AQP4	[85]
	PTE	Male SD rats that received autologous blood injection	↑: SIRT1, Nrf2, OPA1, JC-1 ↓: ROS, MDA, Caspase-3, TUNEL	[88]
		Mouse BV-2 microglial cell line	↑: Nrf2, HO-1, Arg-1, CD206 ↓: iNOS, CD16/32, TNF-α, IL-1β, IL-6, NF-κB p65	[89]
		Male C57BL/6 mice that received collagenase injection	↑: Arg-1, CD206, Nrf2	
	SAA	Male C57BL/6 mice that received autologous blood injection	↑: Nrf2, GPX4, xCT(SLC7A11), GSH ↓: Fe^2+^, ACSL4, MDA, ROS	[90]
		Primary cortical neurons of neonatal C57BL/6 mice	↑: Nrf2, GPX4, xCT ↓: ROS, Fe^2+^	
	Polydatin	Male SD rats that received autologous blood injection	↑: Nrf2, NQO1, HO-1, SOD, GSH, GSSG ↓: NO, MDA, NSE	[94]
Terpenoids	Andrographolide	Male SD rats that received autologous blood injection	↑: Nrf2, HO-1, SOD, CAT ↓: MDA, NF-κB p65, MMP-9, BCSF permeability	[97]
	Crocin	Male C57BL/6 mice that received collagenase injection	↑: SOD, GSH-Px, GPX4, FTH1, SLC7A11 (xCT), Nrf2 ↓: MDA, Fe^2+^	[101]
		Mouse HT22 hippocampal neuron cell line	↑: GPX4, FTH1, SLC7A11, Nrf2 ↓: ROS, Fe^2+^	
Other categories	Phillyrin	Male C57BL/6 mice that received collagenase injection	↑: Nrf2, HO-1, NQO1, SOD-1 ↓: ROS, MDA, Caspase-3, TUNEL	[104]
	Withaferin A	Human-derived SH-SY5Y neuroblastoma cell line	↑: Nrf2, HO-1, GPX4, GSH ↓: Fe^2+^, ROS	[107]
	Betaine	Male C57BL/6 mice that received autologous blood injection	↑: GSH, GPX4, Nrf2, HO-1 ↓: ROS, MDA, ACSL4, Fe^2+^	[111]
		Primary cortical neurons of neonatal C57BL/6 mice	↑: GPX4, Nrf2, GSH ↓: ROS, Fe^2+^, ACSL4	
	Silymarin	Male SD rats injected with type IV collagenase	↑: Nrf2, HO-1 ↓: ROS, MDA, NF-κB p65, NLRP3, Caspase-1, IL-1β	[115]
	Sulforaphane	Experimental ICH model in Nrf2-deficient mice	↑: Nrf2, CAT, SOD, NQO1, GST ↓: 3-NT, 4-HNE	[34]
		Microglial erythrocyte-phagocytosis model and experimental ICH model in wild-type/Nrf2-knockout mice	↑: Nrf2, GST, SOD1, NQO1	[8]

Note: In vitro hemin-, hemoglobin-, oxyhemoglobin-, or cell-based models were considered ICH-related mechanistic models but were not treated as equivalent to in vivo ICH efficacy evidence. Evidence from non-ICH models is explicitly identified where applicable and was used only as indirect mechanistic support.

**Table 3:** Evidence grading of Nrf2-related validation for plant-derived natural compounds in experimental ICH.

Class	Compound	Nrf2-Related Validation	Evidence Level
Flavonoids	Baicalin	Nrf2/HO-1/NQO1/SLC7A11/GPX4 upregulation; Nrf2 inhibitor attenuated protection in one study	Level 1; additional evidence remains Level 2
	Baicalein	Nrf2/ARE activation and antioxidant/anti-inflammatory effects; no clear Nrf2-dependency blockade	Level 2
	Luteolin	p62/Keap1/Nrf2 activation; HO-1/NQO1 upregulation; no direct Nrf2 blockade	Level 2
	EGCG	Nrf2 inhibition or ML385 partially weakened anti-ferroptotic effects; HO-1 inhibitor attenuated pyroptosis-related protection	Level 1
	Epicatechin	Nrf2 activation observed, but EC also regulates AP-1/HO-1 under Nrf2-knockout conditions	Level 2; Nrf2-independent AP-1/HO-1 mechanism also involved
	Isoliquiritigenin	Nrf2 gene silencing abolished protective effects	Level 1
	Kaempferol	AKT/Nrf2/HO-1 activation; no direct Nrf2 blockade	Level 2
	Calycosin	Nrf2 upregulation with NF-κB/NLRP3 suppression; no direct Nrf2-dependency validation	Level 2
Phenolics	Resveratrol	ICH efficacy evidence; Nrf2-causality evidence partly from non-ICH models	Level 2 for ICH efficacy; Level 3 for Nrf2-causality evidence
	Gastrodin	Keap1/Nrf2/HO-1 activation; no direct Nrf2 blockade	Level 2
	Curcumin/Curcumin-loaded nanoparticles	Nrf2/HO-1/NQO1/GPX4 upregulation; no clear Nrf2-dependency blockade	Level 2
	Protocatechuic acid	Nrf2 knockdown attenuated HO-1, ZO-1, and occludin upregulation	Level 1; additional evidence remains Level 2
	Pterostilbene	SIRT1 inhibition supports upstream pathway involvement; no direct Nrf2 blockade; OPA1-dependent mechanism also involved	Level 2
	Salvianolic acid A	AKT inhibitor and ML385 reversed anti-ferroptotic effects in ICH-related models	Level 1
	Polydatin	Nrf2/NQO1/HO-1 upregulation; no direct Nrf2 blockade	Level 2
Terpenoids	Andrographolide	Keap1/Nrf2/HO-1 activation; no direct Nrf2-dependency blockade	Level 2
	Crocin	Nrf2 nuclear translocation with GPX4/FTH1/SLC7A11 upregulation; no direct Nrf2 blockade	Level 2
Other	Phillyrin	ML385 reversed antioxidant and anti-apoptotic effects	Level 1
	Withaferin A	Nrf2 silencing or HO-1 inhibition reversed protective effects	Level 1
	Betaine	Nrf2 inhibition weakened anti-edematous, antioxidant, and anti-ferroptotic effects	Level 1
	Silymarin	Nrf2/HO-1 upregulation with NF-κB/NLRP3 suppression; no direct Nrf2-dependency blockade	Level 2
	Sulforaphane	Protective effects were abolished in Nrf2-deficient mice; hematoma-clearance effect was lost in Nrf2-knockout mice	Level 1

Note: Level 1 indicates causal Nrf2-dependent evidence supported by genetic or pharmacological pathway blockade. Level 2 indicates Nrf2-associated evidence without direct validation of Nrf2 dependency. Level 3 indicates indirect or context-transferred evidence mainly derived from non-ICH models. For compounds with mixed evidence, the level reflects the strongest ICH-related evidence while noting limitations in causal validation and translational relevance. For compounds with both causal and associative studies, the highest evidence level is reported, while lower-level supporting evidence is noted separately.

**Table 4:** Operational prioritization matrix integrating Nrf2 evidence strength and translational readiness of plant-derived natural compounds in experimental ICH.

Compound	D1: ICH Efficacy	D2: Nrf2 Causality	D3: Brain Exposure/PK Feasibility	D4: Therapeutic/Model Relevance	D5: Long-Term/Safety Evidence	Total /10	Priority	Key Reason for Prioritization or Downgrading
Baicalin	2	2	0	1	1	6	Moderate	Partial causal Nrf2 validation is available, because Nrf2 inhibition attenuated protective effects in one ICH-related study, whereas additional evidence remains associative. However, absence of perihematomal brain concentration data and limited long-term safety evidence reduce translational priority.
EGCG	2	2	0	1	0	5	Moderate	ML385 partially attenuated anti-ferroptotic effects and HO-1 inhibition attenuated pyroptosis-related protection, supporting pharmacological involvement of Nrf2/HO-1-related signaling. However, partial rather than complete abrogation limits causal confidence, and uncertain brain exposure plus known pharmacokinetic barriers prevent higher priority.
Isoliquiritigenin	2	2	0	1	0	5	Moderate	Nrf2 gene silencing abolished protective effects, providing strong causal evidence in the cited ICH model. Translational priority is limited by undefined pharmacokinetics, brain exposure, dose-response relationship, therapeutic window, and long-term safety profile.
Protocatechuic acid	2	2	0	1	0	5	Moderate	Nrf2 knockdown attenuated HO-1, ZO-1, and occludin upregulation, supporting causal pathway involvement in BBB protection. Priority is limited by lack of brain concentration data, delayed-treatment studies, and long-term outcome data.
Salvianolic acid A	2	2	0	1	0	5	Moderate	AKT inhibition and ML385 reversed anti-ferroptotic effects, supporting functional involvement of AKT/Nrf2-related signaling. Priority is limited by absence of perihematomal brain exposure data, reliance on standard young healthy rodent models, and lack of delayed-treatment or long-term outcome studies.
Phillyrin	2	2	0	1	0	5	Moderate	ML385 reversal supports functional Nrf2 involvement in antioxidant and anti-apoptotic effects. Priority is limited by undefined pharmacokinetics, BBB penetration, brain exposure, delayed-treatment efficacy, and long-term functional outcomes.
Betaine	2	2	0	1	0	5	Moderate	Nrf2 inhibition substantially weakened neuroprotective, anti-edematous, and anti-ferroptotic effects, supporting causal Nrf2 involvement. Priority is limited by absence of perihematomal brain concentration data, and the long-term safety implications of methyl-donor activity require further investigation.
Sulforaphane	2	2	0	1	0	5	Moderate	Strong Nrf2-dependent evidence is available, including loss of antioxidant and hematoma-clearance effects in Nrf2-deficient or Nrf2-knockout mice. However, perihematomal brain exposure, delayed-treatment validation, clinically relevant comorbidity models, and long-term safety evidence remain insufficient.
Gastrodin	2	1	1	1	0	5	Moderate-low	Relatively favorable water solubility and CNS distribution reported in non-ICH settings provide indirect pharmacokinetic support. However, no Nrf2 blockade experiment has been performed in ICH models; evidence remains Nrf2-associated. Perihematomal brain concentration and delayed-treatment efficacy remain uncharacterized.
Curcumin/curcumin-loaded nanoparticles	2	1	1	1	0	5	Moderate-low	Platelet membrane-coated PLGA nanoparticles improve delivery rationale and provide a potential strategy for overcoming curcumin’s severe pharmacokinetic limitations. However, Nrf2 causality remains unvalidated, and effective perihematomal exposure even with nanoformulation remains unconfirmed.
Pterostilbene	2	1	1	1	0	5	Moderate-low	More favorable pharmacokinetic properties than resveratrol in non-ICH settings improve translational rationale. SIRT1 inhibition supports upstream pathway involvement, but no direct Nrf2 blockade has been performed in ICH models. The OPA1-dependent mechanism is Nrf2-independent, and specific Nrf2 dependency remains unconfirmed.
Baicalein	2	1	0	1	0	4	Low	Evidence supports Nrf2/ARE activation and antioxidant or anti-inflammatory effects in ICH-related models, but no Nrf2-dependency blockade experiment has been reported. Rapid metabolism and absence of brain exposure data further limit translational priority.
Luteolin	2	1	0	1	0	4	Low	p62/Keap1/Nrf2 pathway activation is reported, but no direct Nrf2 blockade experiment has been performed. TLR4/TRAF6/NF-kappaB inhibition appears at least partly Nrf2-independent. Brain exposure and therapeutic window remain undefined.
Kaempferol	2	1	0	1	0	4	Low	AKT/Nrf2/HO-1 activation is associated with reduced oxidative stress and apoptosis, but no Nrf2 knockout, knockdown, selective inhibition, or rescue experiment has been reported. Brain exposure and clinically relevant model validation are lacking.
Calycosin	2	1	0	1	0	4	Low	Nrf2-related antioxidant and anti-inflammatory changes are reported, but causal Nrf2 dependency remains unvalidated. Brain exposure and therapeutic window are undefined; current evidence remains mainly associative.
Resveratrol	2	1	0	1	0	4	Low	ICH efficacy evidence is available, but Nrf2-causality evidence is derived mainly from non-ICH models and should be treated as indirect mechanistic support. Pharmacokinetic constraints and unconfirmed brain exposure in ICH further limit prioritization.
Polydatin	2	1	0	1	0	4	Low	Nrf2/NQO1/HO-1 upregulation is associative, with no reported Nrf2 blockade experiment. Dependence on metabolic conversion and uncharacterized brain exposure limit translational confidence.
Andrographolide	2	1	0	1	0	4	Low	Keap1/Nrf2/HO-1 activation is reported, but no Nrf2 blockade experiment has been performed. Poor aqueous solubility, limited CNS exposure data, unverified target engagement, and absent brain concentration data restrict translational potential.
Crocin	2	1	0	1	0	4	Low	Anti-ferroptotic effects are accompanied by Nrf2 nuclear translocation and downstream upregulation, but no Nrf2 blockade experiment has been reported. High polarity may limit passive BBB diffusion, and brain concentration in ICH remains uncharacterized.
Silymarin	2	1	0	1	0	4	Low	Nrf2/HO-1 upregulation with NF-κB/NLRP3 suppression is reported, but direct Nrf2-dependency validation is lacking. Poor oral bioavailability, heterogeneous flavonolignan composition, and absent brain exposure data prevent higher priority.
Withaferin A	1	2	0	0	0	3	Low	Nrf2 silencing and HO-1 inhibition reversed neuroprotection, providing causal mechanistic support. However, evidence is entirely from an in vitro hemin-induced neuronal model; no in vivo ICH validation, brain exposure, dosing, safety, or therapeutic-window data are available.
Epicatechin	1	1	0	0	0	2	Low	The mechanism involves both Nrf2-associated antioxidant responses and Nrf2-independent AP-1/HO-1 regulation. Current evidence is insufficient to support Nrf2-dependent neuroprotection; Nrf2-independent HO-1 suppression may be the more relevant mechanism for reducing iron-mediated injury after ICH.

Note: To further operationalize translational appraisal, we constructed a compound-prioritization matrix that converts key limitations—including lack of brain concentration data, absence of delayed-treatment testing, use of young healthy animal models, and lack of long-term safety assessment—into explicit downgrading factors. Each compound was scored across five dimensions: D1, ICH efficacy; D2, Nrf2 causality; D3, brain exposure/PK feasibility; D4, therapeutic/model relevance; and D5, long-term/safety evidence. Each dimension was scored from 0 to 2 points, with a maximum total score of 10. Compounds were ranked by total score and then interpreted according to the priority categories defined below. Priority interpretation: Low priority was defined as 0–4 points or in vitro-only evidence. Moderate-low priority was defined as 5 points without direct Nrf2 causal validation but with indirect pharmacokinetic or delivery rationale. Moderate priority was defined as 5–7 points with direct or partial Nrf2 causal validation. Relatively high priority was defined as ≥8 points. However, classification as relatively high priority required not only Nrf2-related mechanistic evidence but also delayed-treatment testing, brain-exposure support, clinically relevant model validation, and long-term safety or functional evidence. Important note: No compound currently reaches relatively high priority (≥8 points), because direct perihematomal brain concentration data in ICH models are largely unavailable. The uniformly low scores for long-term/safety evidence also reflect a systematic gap across the field rather than a compound-specific limitation. For compounds without brain exposure data, translational priority is effectively capped at moderate even when causal Nrf2 evidence is available. In vitro-only evidence caps priority at low regardless of Nrf2 causality strength. Scoring criteria for Table 4: D1, ICH-specific efficacy: 0 = no direct ICH efficacy evidence or evidence derived mainly from non-ICH models; 1 = in vitro ICH-related evidence or limited in vivo ICH evidence based mainly on molecular or biochemical endpoints; 2 = in vivo ICH animal-model evidence showing improvement in neurological, histological, edema, hematoma, or functional outcomes. D2, Nrf2 causality: 0 = no Nrf2-related endpoint or only indirect pathway inference; 1 = increased Nrf2 nuclear translocation or upregulation of Nrf2 downstream targets without Nrf2-dependency validation; 2 = Nrf2 knockout, knockdown, selective inhibition, or rescue-based validation showing significant attenuation or reversal of key protective effects. When quantitative effect sizes were available, ≥50% reversal of compound-induced protection after Nrf2 blockade was considered strong Nrf2-dependent evidence, whereas statistically significant but <50% reversal was considered partial Level 1 evidence and was downgraded in prioritization. D3, brain exposure and pharmacokinetic feasibility: 0 = no brain-exposure or pharmacokinetic information; 1 = indirect pharmacokinetic evidence, CNS distribution data from non-ICH settings, or delivery strategies suggesting potential brain exposure; 2 = measured brain, cerebrospinal fluid, or perihematomal concentrations, preferably linked to pharmacodynamic Nrf2 activation or downstream target engagement. D4, therapeutic window and clinically relevant model design: 0 = pretreatment, prophylactic treatment, or treatment initiated only at ≤1 h after ICH in young healthy animals; 1 = treatment initiated at ≥3 h after ICH or inclusion of one clinically relevant model feature, such as aged, female, hypertensive, anticoagulated, or comorbidity models; 2 = treatment initiated at ≥6–24 h after ICH and validated in at least one clinically relevant model feature, such as aged, female, hypertensive, anticoagulated, or comorbidity conditions. D5, long-term outcome and safety evidence: 0 = acute endpoints only, generally ≤7 days, with no systematic safety assessment; 1 = subacute functional or histological outcomes with limited safety assessment; 2 = ≥28-day neurological or histological outcomes combined with systematic safety assessment. Systematic safety assessment included, when relevant, liver and renal toxicity, hematological indices, platelet/coagulation parameters, hematoma expansion, blood pressure effects, and potential drug–drug interactions with antihypertensive agents, anticoagulation reversal therapies, or other medications commonly used in ICH patients.

## 6. Discussion and Perspectives

In summary, the Nrf2 signaling pathway should not be regarded merely as a single antioxidant response pathway, but as an integrative signaling node linking oxidative stress, iron handling, ferroptosis, neuroinflammation, blood–brain barrier injury, and hematoma clearance after ICH [7]. Hematoma degradation induces ROS generation, iron accumulation, lipid peroxidation, inflammasome activation, neurovascular injury, and impaired phagocytic clearance, forming a multistage and multicellular secondary injury network [33]. Within this context, Nrf2 provides a biologically plausible framework for evaluating secondary brain injury after ICH. However, pathway activation alone should not be interpreted as therapeutic causality.

The plant-derived natural compounds reviewed here show marked diversity in chemical structures, pharmacological profiles, and evidence strength. Many compounds are associated with Keap1/Nrf2, p62/Keap1/Nrf2, Akt/Nrf2, SIRT1/Nrf2, or Nrf2/HO-1-related signaling, and may influence oxidative stress, ferroptosis, inflammation, BBB integrity, and hematoma resolution. Nevertheless, these compounds should not be treated as a uniform group of Nrf2-dependent neuroprotective agents. As summarized in Table 3, only a limited subset has been supported by genetic or pharmacological validation of Nrf2 dependency, whereas many studies remain based on Nrf2-associated molecular changes.

A major limitation of the current evidence is its low translational relevance. Most studies have used young, healthy, predominantly male rodents and collagenase- or autologous blood-induced ICH models, which cannot fully reproduce the clinical context of advanced age, hypertension, diabetes, cerebral amyloid angiopathy, anticoagulant exposure, and multimorbidity [117,118]. In addition, many compounds were administered before or immediately after ICH induction, whereas clinical intervention is usually delayed. Beyond model design and treatment timing, ICH-specific risk–benefit assessment should also consider potential platelet and coagulation effects, particularly for polyphenols or other plant-derived compounds with reported antiplatelet, anticoagulant, vasodilatory, or blood-pressure-modulating properties. Therefore, the absence of overt toxicity should not be interpreted as sufficient safety evidence; hematoma expansion, platelet/coagulation parameters, blood pressure effects, and clinically relevant drug–drug interactions should be evaluated when appropriate. Future studies should also use aged animals, comorbidity models, both sexes, delayed therapeutic windows, randomization, blinded assessment, sample size estimation, ICH-specific safety assessment, and long-term functional outcomes [119]. Recent international Nrf2 research initiatives have further emphasized that the translational interpretation of Nrf2-targeted interventions depends on appropriate model selection, genetic or pharmacological validation of Nrf2 modulation, and clinically relevant endpoints [120]. This consideration is directly relevant to ICH, because hemorrhage-specific processes, including hemoglobin toxicity, iron overload, perihematomal inflammation, and hematoma clearance, are not fully reproduced by non-ICH models.

Botanical candidates for ICH require additional translational scrutiny beyond efficacy in preclinical models. First, batch-to-batch standardization remains a major practical issue for plant-derived compounds, especially when crude extracts or multi-component formulations are used. Future studies should report the botanical source, extraction method, chemical fingerprint, purity, content of the proposed active compound, and contaminant testing. Second, compound-specific toxicological risks should be incorporated into translational appraisal. For example, concentrated green tea extracts and high-dose EGCG-containing products have been associated with liver injury in susceptible individuals, indicating that antioxidant activity should not be equated with systemic safety. Third, ICH-specific bleeding risk must be considered. Some polyphenols, including resveratrol, may affect platelet function, coagulation pathways, or drug metabolism, and high-dose resveratrol has been reported to enhance warfarin anticoagulant activity in experimental settings. This issue is particularly relevant for ICH patients, who frequently receive antihypertensive agents, anticoagulation reversal therapies, antithrombotic discontinuation, or intensive-care medications. Therefore, future studies should evaluate not only neurological and molecular endpoints but also liver enzymes, platelet count and function, coagulation parameters, blood pressure effects, and interactions with clinically relevant ICH medications.

A central message of this review is that Nrf2 association should not be equated with Nrf2 dependence. Increased Nrf2 nuclear translocation or upregulation of HO-1, NQO1, GPX4, SLC7A11, or related targets supports pathway involvement but does not prove that Nrf2 is required for neuroprotection. Future studies should incorporate Nrf2 knockout, cell-type-specific Nrf2 deficiency, siRNA/shRNA-mediated knockdown, selective inhibitors such as ML385, rescue experiments, ARE reporter assays, and target-engagement analyses. Such approaches are needed to distinguish direct Nrf2-dependent mechanisms from indirect antioxidant or anti-inflammatory effects.

The context-dependent effects of Nrf2 signaling also require careful consideration. HO-1 illustrates this complexity: moderate early induction may facilitate heme degradation and antioxidant protection, whereas sustained or excessive HO-1 activation may increase free iron release and aggravate lipid peroxidation and ferroptosis [28]. Therefore, future work should define the optimal timing, magnitude, duration, and cell-type specificity of Nrf2 activation in neurons, microglia, astrocytes, endothelial cells, and infiltrating macrophages.

A critical but frequently underappreciated barrier to translation is whether the reviewed compounds reach therapeutically relevant concentrations in perihematomal brain tissue. Across the compounds discussed, no study has reported direct perihematomal brain concentration measurements in ICH models, representing a critical translational gap that substantially limits conclusions about lesion-site target engagement and effective dosing. Several compounds, including curcumin, resveratrol, and EGCG, are known to face substantial pharmacokinetic constraints, such as limited aqueous solubility, poor systemic bioavailability, rapid phase I/II metabolism, conjugation, or chemical instability [54,55,71,72,79,80]. These limitations make it difficult to determine whether the doses used in preclinical ICH studies produce sufficient local exposure at the hemorrhagic lesion site. In contrast, compounds such as pterostilbene and gastrodin may have relatively more favorable pharmacokinetic properties in non-ICH settings [76,77,87]. However, systemic bioavailability does not necessarily indicate adequate perihematomal brain exposure, and this distinction is critical for translational appraisal. Therefore, future studies should integrate pharmacodynamic endpoints with plasma concentrations, brain tissue concentrations, metabolite profiling, target engagement, dose–response relationships, and therapeutic-window analysis. For compounds with stronger causal evidence, such as ILG, PCA, SAA, phillyrin, WFA, and BET, the next priority should be to determine effective brain exposure and delayed-treatment efficacy in clinically relevant ICH models. For compounds supported mainly by Nrf2-associated evidence, pharmacokinetic characterization and brain exposure assessment should accompany, or even precede, further mechanistic testing. Delivery approaches, such as platelet membrane-coated curcumin-loaded PLGA nanoparticles, provide one possible strategy for improving lesion-targeted accumulation and overcoming BBB-related limitations [82].

Overall, plant-derived natural compounds currently provide mechanistic hypotheses and preliminary experimental leads for investigating Nrf2-related signaling in ICH, rather than established Nrf2-dependent therapeutic candidates. As shown in Table 4, no compound currently reaches relatively high translational priority because direct perihematomal brain exposure and long-term safety evidence are largely unavailable. Therefore, future research should first demonstrate Nrf2 target dependence, measurable lesion-site exposure, delayed-treatment efficacy, and sustained functional benefit before these compounds can be considered for translational development.

## 7. Conclusions

This review critically evaluated plant-derived natural compounds targeting Nrf2-centered redox signaling in experimental ICH. Current evidence supports the biological relevance of Nrf2 in secondary brain injury, particularly in oxidative stress, ferroptosis, neuroinflammation, BBB disruption, and hematoma clearance. However, the strength of evidence varies substantially among compounds. Only a limited number of studies have provided causal validation using Nrf2 inhibition, knockdown, or related pathway-blockade approaches, whereas many studies remain based on Nrf2-associated molecular changes.

Therefore, for most plant-derived natural compounds, current evidence does not yet support definitive Nrf2-dependent neuroprotection in ICH. These compounds should be regarded primarily as hypothesis-generating mechanistic leads unless causal Nrf2 validation, measurable perihematomal brain exposure, delayed therapeutic efficacy, and long-term functional benefit are demonstrated. A shift from pathway observation to mechanism validation and operational translational prioritization is essential for future research.

## Data Availability

Data sharing is not applicable to this article, as no new data were created or analyzed in this study. All information discussed is available from the publications cited in the reference list.

## Abbreviations

The following abbreviations are used in this manuscript:

ICH	Intracerebral hemorrhage
SBI	Secondary brain injury
Nrf2	Nuclear factor erythroid 2-related factor 2
Keap1	Kelch-like ECH-associated protein 1
ARE	Antioxidant response element
HO-1	Heme oxygenase-1
NQO1	NAD(P)H quinone oxidoreductase 1
SOD	Superoxide dismutase
GPX4	Glutathione peroxidase 4
SLC7A11	Solute carrier family 7-member 11
xCT	Alias of SLC7A11
PI3K	Phosphatidylinositol 3-kinase
Akt	Protein kinase B
SIRT1	Sirtuin 1
ROS	Reactive oxygen species
DAMPs	Damage-associated molecular patterns
BBB	Blood–brain barrier
NVU	Neurovascular unit
bZIP	Basic leucine zipper
sMafs	Small Maf proteins
GCLC	Glutamate-cysteine ligase catalytic subunit
GSH	Glutathione
GSSG	Glutathione disulfide
Trx	Thioredoxin
TrxR	Thioredoxin reductase
3-NT	3-nitrotyrosine
4-HNE	4-hydroxynonenal
SRA	Scavenger receptor A
TLR4	Toll-like receptor 4
MyD88	Myeloid differentiation factor 88
NF-κB	Nuclear factor-κB
IL-1β	Interleukin-1β
TNF-α	Tumor necrosis factor-α
IL-6	Interleukin-6
NLRP3	NOD-like receptor family pyrin domain containing 3
CBP	CREB-binding protein
TXNIP	Thioredoxin-interacting protein
ZO-1	Zonula occludens-1
MMP	Matrix metalloproteinase
ACSL4	Acyl-CoA synthetase long-chain family member 4
EGCG	Epigallocatechin-3-gallate
EC	(-)-Epicatechin
ILG	Isoliquiritigenin
PTE	Pterostilbene
SAA	Salvianolic acid A
BET	Betaine
SIL	Silymarin
mNSS	Modified neurological severity scores
NSE	Neuron-specific enolase
MDA	Malondialdehyde
AQP4	Aquaporin 4
OPA1	Optic atrophy 1
iNOS	Inducible nitric oxide synthase
Arg-1	Arginase-1
FTH1	Ferritin heavy chain 1
Lcn2	Lipocalin 2
GST	Glutathione S-transferase
GR	Glutathione reductase
CAT	Catalase
PLGA	Poly (lactic-co-glycolic acid)
PCNPs	Platelet membrane-coated curcumin-loaded PLGA nanoparticles
GSDMD	Gasdermin D
GSDMD-N	N-terminal fragment of gasdermin D
8-OHdG	8-hydroxy-2′-deoxyguanosine
Bcl-2	B-cell lymphoma 2
Bax	Bcl-2-associated X protein
TUNEL	Terminal deoxynucleotidyl transferase dUTP nick end labeling
SFN	Sulforaphane
